# Whole genome sequencing and de novo assembly identifies Sydney-like variant noroviruses and recombinants during the winter 2012/2013 outbreak in England

**DOI:** 10.1186/1743-422X-10-335

**Published:** 2013-11-13

**Authors:** T H Nicholas Wong, Bethany L Dearlove, Jessica Hedge, Adam P Giess, Paolo Piazza, Amy Trebes, John Paul, Erasmus Smit, E Grace Smith, Julian K Sutton, Mark H Wilcox, Kate E Dingle, Tim E A Peto, Derrick W Crook, Daniel J Wilson, David H Wyllie

**Affiliations:** 1 Nuffield Department of Medicine, John Radcliffe Hospital, University of Oxford, Oxford, UK; 2 Oxford NIHR Biomedical Research Centre, John Radcliffe Hospital, Oxford, UK; 3 Wellcome Trust Centre for Human Genetics, Roosevelt Drive, Oxford, UK; 4 Public Health England Collaborating Centre, Oxford; John Radcliffe Hospital, Oxford, UK; 5 Public Health Laboratory, Royal Sussex County Hospital, Brighton, UK; 6 Public Health Laboratory, Heart of England NHS Foundation Trust, Birmingham, UK; 7 Public Health Laboratory, Southampton General Hospital, Southampton, UK; 8 Public Health Laboratory, Leeds Teaching Hospitals NHS Trust, Leeds, UK

**Keywords:** Norovirus, Outbreaks, Whole genome sequencing, Calicivirus, Gastroenteritis, Infection control

## Abstract

**Background:**

Norovirus is the commonest cause of epidemic gastroenteritis among people of all ages. Outbreaks frequently occur in hospitals and the community, costing the UK an estimated £110 m per annum. An evolutionary explanation for periodic increases in norovirus cases, despite some host-specific post immunity is currently limited to the identification of obvious recombinants. Our understanding could be significantly enhanced by full length genome sequences for large numbers of intensively sampled viruses, which would also assist control and vaccine design. Our objective is to develop rapid, high-throughput, end-to-end methods yielding complete norovirus genome sequences. We apply these methods to recent English outbreaks, placing them in the wider context of the international norovirus epidemic of winter 2012.

**Method:**

Norovirus sequences were generated from 28 unique clinical samples by Illumina RNA sequencing (RNA-Seq) of total faecal RNA. A range of *de novo* sequence assemblers were attempted. The best assembler was identified by validation against three replicate samples and two norovirus qPCR negative samples, together with an additional 20 sequences determined by PCR and fractional capillary sequencing. Phylogenetic methods were used to reconstruct evolutionary relationships from the whole genome sequences.

**Results:**

Full length norovirus genomes were generated from 23/28 samples. 5/28 partial norovirus genomes were associated with low viral copy numbers. The *de novo* assembled sequences differed from sequences determined by capillary sequencing by <0.003%. Intra-host nucleotide sequence diversity was rare, but detectable by mapping short sequence reads onto its *de novo* assembled consensus. Genomes similar to the Sydney 2012 strain caused 78% (18/23) of cases, consistent with its previously documented association with the winter 2012 global outbreak. Interestingly, phylogenetic analysis and recombination detection analysis of the consensus sequences identified two related viruses as recombinants, containing sequences in prior circulation to Sydney 2012 in open reading frame (ORF) 2.

**Conclusion:**

Our approach facilitates the rapid determination of complete norovirus genomes. This method provides high resolution of full norovirus genomes which, when coupled with detailed epidemiology, may improve the understanding of evolution and control of this important healthcare-associated pathogen.

## Background

Norovirus is a leading cause of diarrhoea worldwide and the most common aetiological agent of epidemic gastroenteritis in all age groups [1], being recognised in semi-closed institutional settings, particularly hospitals and care centres for the elderly, child care centres, military settings and cruise ships [2,3]. The cost and burden of norovirus outbreaks on the use of health resources is substantial [4], hence rapid and effective interventions to reduce norovirus transmission are needed. However, successful development of these interventions relies, in part, on improving our understanding of norovirus transmission dynamics and global epidemiology.

Multiple norovirus genogroups and genotypes exist. Although co-circulation of different genotypes occurs every winter season, one genotype (GII.4) has dominated over all the others since the early 1990s worldwide, and has been responsible for the majority of outbreaks in the last 20 years [5,6]. Since 2002, norovirus epidemiology has been characterized by ‘winter peaks’ of infection. However, autumn 2012 saw a marked departure from this pattern, with a large and unusually early outbreak affecting Western Europe [7-9]. Molecular typing of reported outbreaks indicated that this was due to a new strain designated Sydney 2012, which largely displaced the previously widespread New Orleans 2009 strain. The Sydney 2012 strain was described first in Australia in March 2012, and later worldwide including in the UK [7,9].

Full length genome sequencing is improving our understanding of both host pathogen relationships and transmission within outbreaks of bacterial [10,11] and viral [12] disease. To address this need for RNA viruses, we recently reported a flexible method using a modified RNA-Seq approach for sequencing direct extracts of faeces [13], yielding full length norovirus genomes by mapping to a reference norovirus sequence. This technique is simpler and likely to be less vulnerable to primer-induced biases than the re-sequencing of single long PCR products, or of multiple smaller PCR products [14]. However, limitations arise from sole reliance on a mapping based approach. In particular, assembling recombinant chimeric viruses, which have recently been reported for Norovirus [15,16], is likely to be incomplete or ineffective, and a *de novo* assembly approach might be more robust. To date, however, using *de novo* assembly of sequences derived from faecal extracts consisting of highly heterogeneous short reads from diverse organisms has not been reported.

Here we describe an end-to-end method for efficient sequencing and *de novo* assembly of full length norovirus genomes building on our RNA-Seq approach, including a comparison of *de novo* assembler performance. We demonstrate the power and future potential of this approach by characterizing the whole genome sequences of multiple GII.4 noroviruses representing October 2012 - January 2013 outbreaks from 10 sites in England and the island of Jersey. We discuss the general applicability of our approach to other viruses, and reflect on the implications of our results for the recent evolution and biological behaviour of the globally transmitted Sydney-like noroviruses.

## Results

### Samples

**Figure 1:**
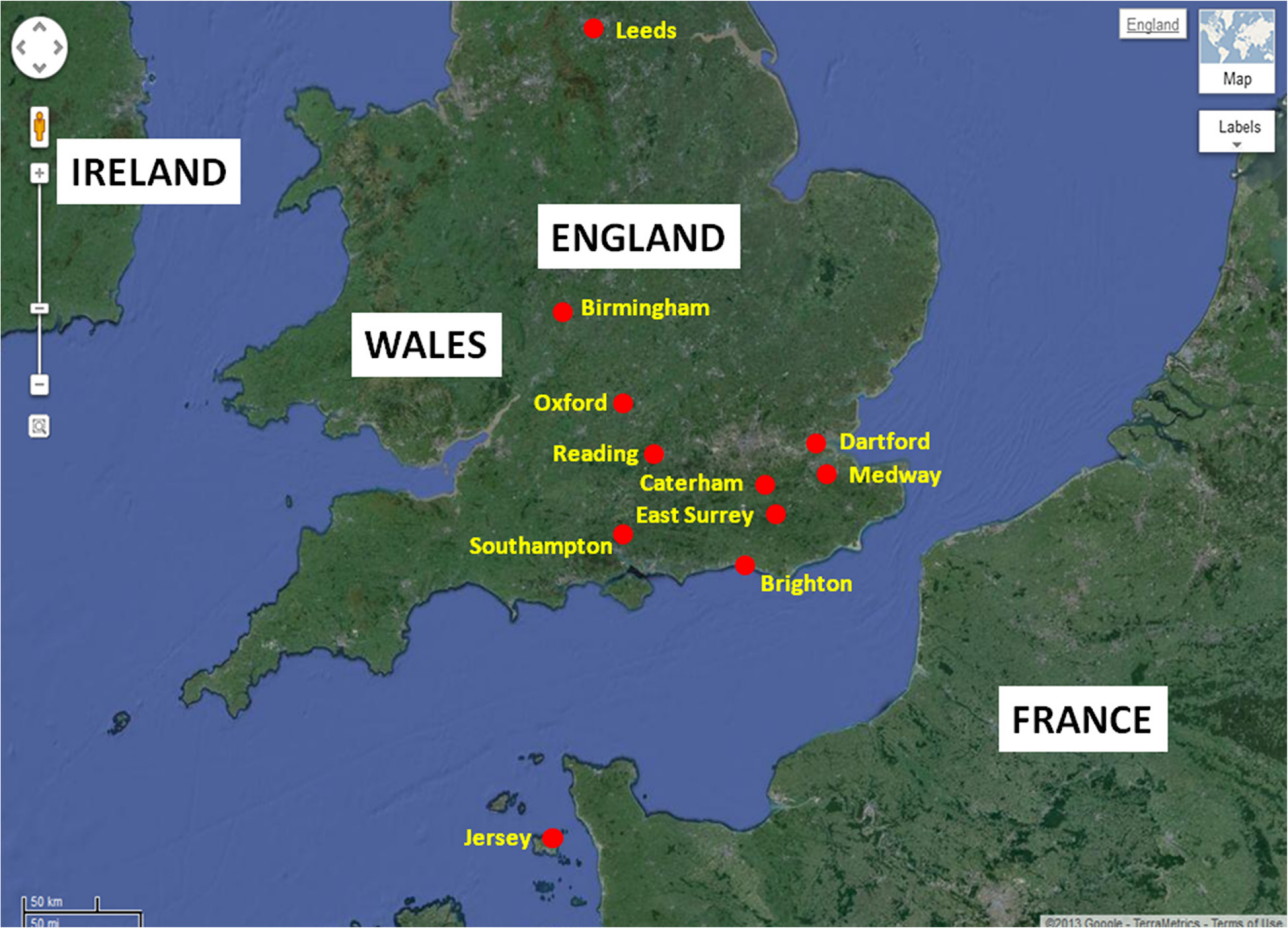
**Norovirus epidemiology and sampling frame.** Locations from which samples were obtained with England and Jersey (map courtesy of Google map).

We collected samples from hospital and community outbreaks in eleven geographically widespread locations between October 2012 and January 2013, while norovirus activity was occurring unusually early in the season (Figure 1). Samples used for sequencing were derived from symptomatic cases and confirmed with quantitative polymerase chain reaction (qPCR) [17]. Viral copies ranged from 10^3^ to 10^8^ copies/μl, similar to samples obtained in winter 2010/2011 outbreaks (mean log_10_ copy number for 2013 5.71 vs. 6.13, p = 0.25 by *t*-test). Illumina MiSeq was utilised for sequencing of all 2012/2013 samples, yielding an average of 2.0 × 10^6^ (range 0.8 × 10^6^ to 3.5 × 10^6^) reads.

### Comparison with known norovirus genomes

Using blastn, we determined the number of reads similar to known human and animal norovirus sequences for each sample studied. We also examined the positions along the genome at which these reads mapped. We observed similar patterns for all sample and known norovirus sequence combinations, with more reads mapping towards the 3′ end of the virus and marked variation in read density across the genome. Counting numbers of reads similar to known norovirus sequences suggested many samples in our study contained sequences most similar to the Sydney norovirus Hu/GII.4/Sydney/NSW0514/2012/AU (Genbank accession JX459908.1); up to 22% of the norovirus reads in some samples matched this genome. One interesting aspect was that while some were similar to the Sydney 2012 strain throughout their length (e.g. samples C00014384 and C00014389), in others (e.g. samples C00014386, C00014390) the 5′ end appeared more similar to other known GII.4 strains.

### De novo assembly of norovirus sequences

#### Assembler performance

**Figure 2:**
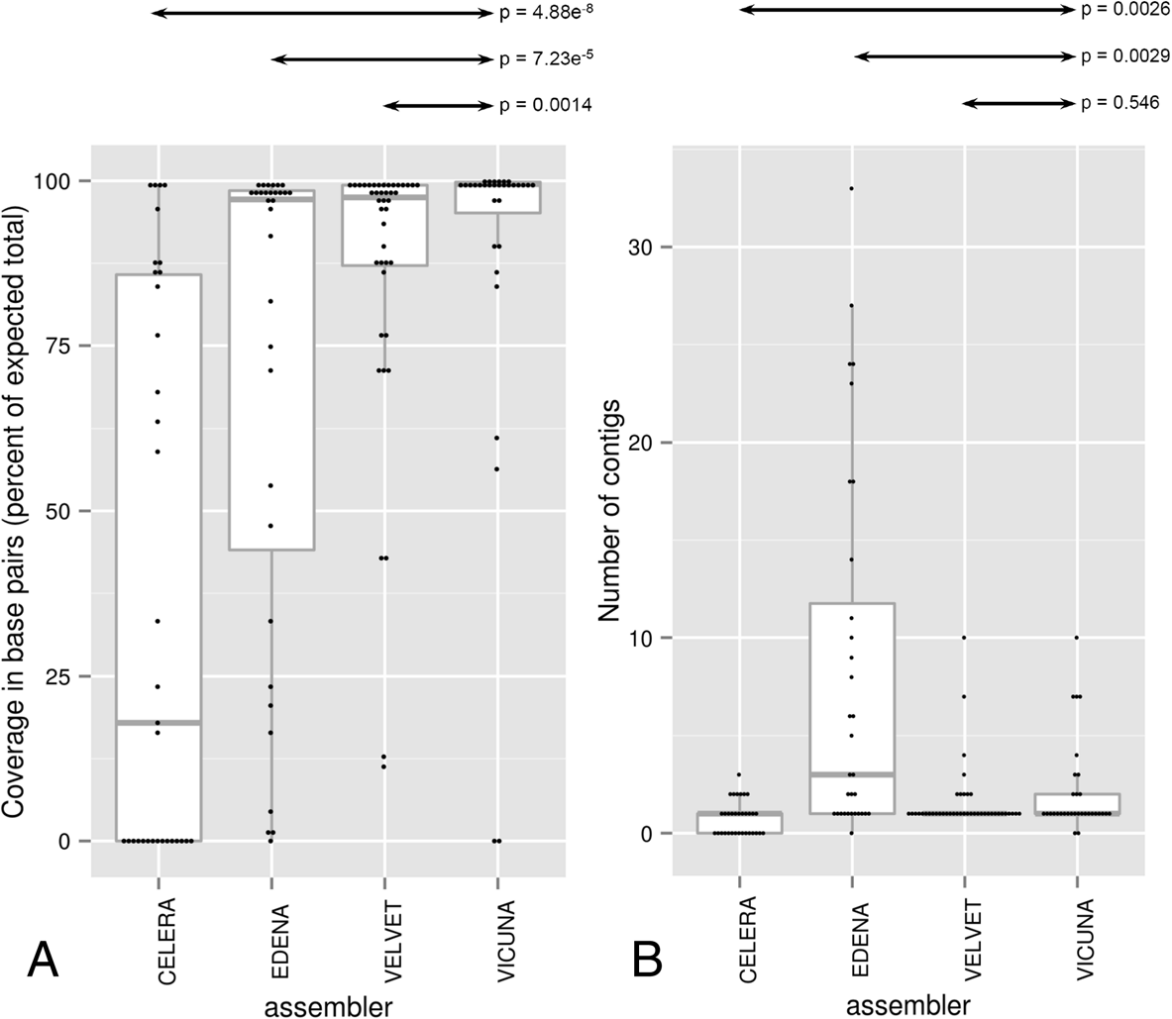
**Assembly performance. (A)** The estimated coverage of the contigs produced and **(B)** the number of contigs produced by four assemblers, on a 32 sample test dataset. One dot signifies the result for one sample. Box and whiskers plots indicating median (thick grey line), 25th and 75th centiles (edges of box) and 5th and 95th centile (ends of whiskers) are shown. Mann–Whitney *U* test are shown comparing each assembler with Vicuna (p values given at the top of each figure).

We compared four assemblers: Velvet [18], which is based on de Bruijn graph construction, with three assemblers (Edena [19], Celera [20] and Vicuna [21]) using various implementations of an alternative strategy, overlap-layout consensus, and a range of parameters. Figure 2A and 2B show assembler performance, having selected the best assembly for each sample/assembler combination. The recently described Vicuna assembler generated the most single-contig assemblies covering >97% (7,321-7,552nt), in 21/32 samples. The best Velvet assemblies were comparable, but the Velvet assembly process was found to be highly sensitive to both k-mer and particularly to the exp_cov parameter, necessitating a large parameter space search to find optimal assemblies (not shown). Vicuna was superior to the other assemblers (Celera, Edena, Velvet) studied (Figure 2A and 2B).

#### Intra-sample diversity

**Figure 3:**
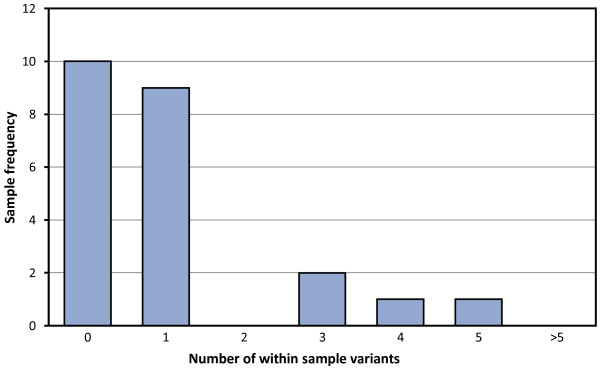
**Intra-sample variation.** Bar plot displaying the frequency of samples and the number of intra-sample variants.

One concern is that the extent of diversity between and within the samples studied might be underestimated by the Vicuna-derived consensus: within sample variation is not revealed. The extent of such intra-sample variation detected is shown in Figure 3. This reveals a limited number of within-host variants in samples that assembled of around one per genome.

#### Determinants of non-assembly

Some samples did not assemble fully. Read numbers did not differ between samples which could not be fully assembled and samples that could be assembled (mean 1.8 × 10^6^ vs. 2.1 × 10^6^ reads (p = 0.22, *t*-test)). In contrast, the total number of reads matching norovirus sequences differed markedly (0.17 × 10^6^ vs. 1.04 × 10^6^ reads, p = 0.009). Read depth appears, therefore, to be a determinant of the ability to assemble using the technique we outlined here. The two qPCR-negative samples did not yield full genomes, although partial assemblies were produced (<59% of the genome in one sample).

#### Reproducibility of the process and relationship to capillary sequencing

To assess the reproducibility of our sequencing and assembly process, one sample was processed and sequenced independently three times starting from the same total RNA extract using the Illumina MiSeq. *De novo* assembly using our method revealed no nucleotide differences between these replicates. We compared the sequences obtained with capillary sequenced isolates (n = 16). We also analysed four repeat samples from a previous Illumina HiSeq run, re-sequenced to confirm reproducibility of both the sequencing and the pipeline analyses. Out of a total of 151,180 bases obtained by capillary sequencing of 20 samples (consisting of 16 samples and 4 replicates), comparison against the *de novo* pipeline generated a total of 49 differences; however, 44 of these were within 13nt of contig termini, suggesting that the two techniques concur well apart from at the very ends of the *de novo* contigs (five variants were not at the contig ends, representing 0.003% discordance between the sequencing methods).

#### Relationship of the outbreak strain to previous viruses

**Figure 4:**
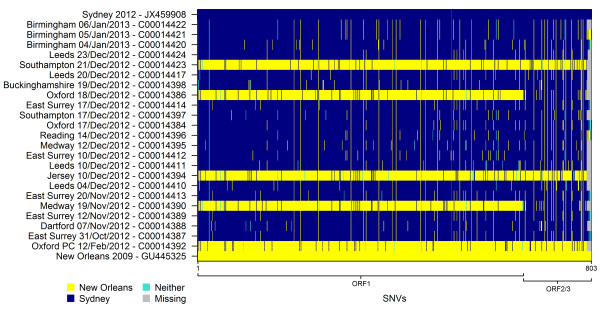
**Graphical SNV matrix.** Graph matrix depicting all 803 single nucleotide variants between New Orleans 2009 and Sydney 2012. Conserved sites within the genome have been removed in this depiction. Nucleotides identical to the Sydney 2012 variant are shown in blue, whilst nucleotides identical to New Orleans 2009 are depicted in yellow. Turquoise areas depict variants that are neither Sydney 2012 nor New Orleans 2009 like. Grey areas depict non assembled information.

**Figure 5:**
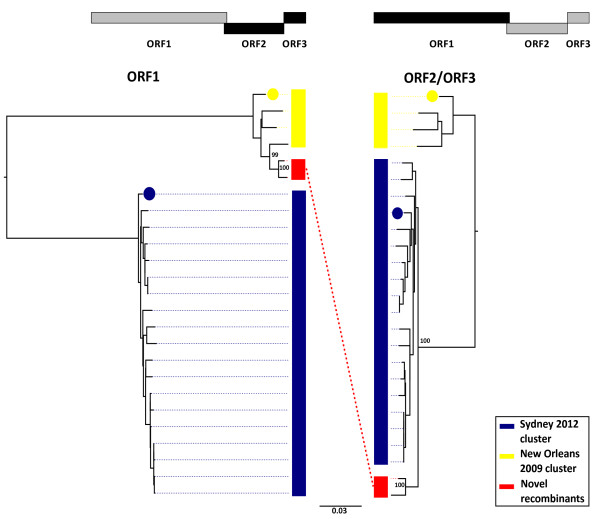
**Norovirus phylogenies for ORF1 and ORF2/3.** Maximum likelihood trees for ORF1 and ORF2/ORF3 regions based on an alignment of 23 norovirus sequences from England and Jersey isolated during winter 2012/13 and two GII.4 reference genomes: New Orleans 2009 and Sydney 2012 (represented with yellow and blue filled circles, respectively). Trees were constructed in PhyML (27) under an HKY85 model of nucleotide substitution, midpoint rooted and bootstrapped with 100 replicates. The bootstrap support is provided for each node (in bold). Clades are coloured according to the reference strain with which they appear to cluster. The dashed line highlights phylogenetic incongruity between ORF1 and ORF2/ORF3, proposed to have arisen by recombination.

Prior to the emergence of the Sydney 2012 strain, the major GII.4 lineage in global circulation was the New Orleans 2009 strain [7,8]. These two strains differ at 803 nucleotide sites (11% of the genome). We investigated the similarity between our dataset of 23 full genome sequences and the two reference sequences at each of these variable sites. For each sequence, Figure 4 shows whether each site is (i) homologous to the Sydney 2012 reference sequence (blue), (ii) homologous to the New Orleans 2009 reference sequence (yellow), or (iii) different to the base in each reference sequence (turquoise). It is clear that three of the 23 genomes show high similarity to the New Orleans 2009 reference strain and 18 show greater similarity to the Sydney 2012 reference strain at the majority of sites analysed. Notably, the sequences similar to Sydney 2012 differ at up to 479 sites (6% of the genome). Figure 4 also identifies two sequences for which the majority of sites are the same as those in New Orleans 2009 (sequences C00014386 and C00014390) although sites towards the end of the genome switch to being more similar to the Sydney 2012 reference strain. This difference implies that these two norovirus samples may represent recombinants with a breakpoint somewhere near the open reading frame (ORF) 1 and ORF2 overlap. This region has been commonly identified as having a high frequency of recombination breakpoints within the GII.4 genotype [15,16]. One of the effects of recombination is that the evolutionary history is not the same across the whole genome, and thus a single tree cannot represent the true ancestry. We therefore investigated the support for recombination at this position by constructing two maximum likelihood phylogenetic trees for ORF1 and ORF2/ORF3 from the 23 whole genome sequences and both New Orleans 2009 and Sydney 2012 reference genomes (Figure 5). The trees show that all 18 Sydney 2012 strains comprise a monophyletic cluster with the Sydney 2012 reference sequence in both ORF1 and ORF2/ORF3 trees. Similarly, the three New Orleans 2009 strains identified in Figure 4 form a separate cluster with the New Orleans 2009 reference sequence in both trees. This clustering is strongly supported in bootstrap analysis (bootstrap values: 92 and 100 in the ORF1 and ORF2/ORF3 trees respectively). The genetic divergence between the Sydney 2012 and New Orleans 2009 clusters is greater in ORF1 than in ORF2/ORF3. The two sequences we identified as potential recombinants in Figure 4 cluster together in both trees, but fall within the New Orleans 2009 cluster in the ORF1 tree and the Sydney 2012 cluster in the ORF2/ORF3 tree. This incongruence in tree topology suggests that these sequences are likely to have originated from a recombination event involving a New Orleans 2009-like ORF1 and Sydney 2012-like ORF2 and 3. Both incongruities in topology between the two trees are illustrated by a dotted line in Figure 5.

We investigated the support for this potential recombination event using the suite of recombination detection methods employed in the software RDP3 (35). No recombination events were detected in any of the 3 New Orleans 2009 strains in the sample (C00014394, C00014416, C00014423). Of the six recombination detection methods used, five provided significant support for the two sequences identified in the maximum likelihood trees as being recombinants. The recombination breakpoint was estimated to be at position 4,971 in the alignment, close to the ORF1/2 overlap (3SEQ p-value = 3.61 × 10^-6^).

Visual inspection of the phylogenetic trees and breakpoint analysis in RDP3 suggests that two recombinant sequences are present in our dataset, most likely sharing the same ancestral recombination event. We suggest that this involved the ORF1 from a New Orleans strain and the ORF2/ORF3 from a close relative of Sydney 2012.

## Discussion

Our study shows that a recently described, primer-independent method of RNA sequencing, together with an optimised bioinformatic pipeline has been established by which complete norovirus genomes can be recovered by *de novo* assembly from ~80% of faecal samples (23/28 qPCR + ve samples). A key part of the bioinformatic pipeline is the use of the Vicuna assembler, which outperformed other assemblers tested.

Since the technique described here is high-throughput, automatable and yields full length norovirus genomes in a high proportion of cases, it has the potential to transform the identification of noroviruses, and to enhance understanding of norovirus transmission. For example, we detected inter-genotypic recombinants, in two out of twenty three samples (~9%), supporting an emerging view that recombination within GII.4 strains may be relatively common. Recombination has been recently recognized to play an important role in the evolution of the GII.4 pandemic lineage [8-12]. The recombination breakpoint identified in our sample (4,971nt) was similar to the recombination hotspot recently described [16]. This further supports the utility of the approach we describe here.

We have shown, using capillary sequencing as a gold standard, that the bioinformatic strategy used (*de-novo* consensus followed by mapping of reads back to the local consensus) produces valid consensus sequences and allows intra-sample strain detection. In theory, there might be individuals with mixed infections in which the *de novo* assembly fails to pick out either consensus. In practice however, such a situation does not appear to have occurred in the samples studied in this paper, where intra-specimen diversity appear to be very limited.

Although the results presented here are encouraging, larger multi-national sample studies are required to fundamentally answer questions about its feasibility in widespread clinical use and infection control. In addition, a larger sampling set may provide the best opportunity to rationalise the depth of coverage required to answer specific questions without wasting potential redundant sequence information.

Our novel end-to-end laboratory and bioinformatic solution generates accurate, complete norovirus genomes in a large majority of clinical samples without any enrichment or sequence-specific PCR steps. As such, it may complement and extend the power of already established epidemiological tools such as Noronet (http://www.rivm.nl/en/Topics/N/NoroNet) which currently rely on low-throughput fractional sequencing small parts of the genome, allowing improved determination of transmission routes in healthcare and community settings, as well as studies of evolution and molecular determinants of pathogenicity.

## Conclusion

We have established an end to end approach using de novo assembly for achieving whole genome sequences of norovirus. This method provides an alternative to current diagnostic tests of norovirus, with the added benefit of supplying detailed resolution of the viral genome and improving our understanding of its evolution and surveillance of this important healthcare associated pathogen.

## Materials and methods

### Samples and laboratory processing8

#### Samples used for de novo assembly analyses

We analysed 46 faeces samples from multiple locations within England and the island of Jersey. These included 28 samples collected from patients with symptoms of diarrhoea and/or vomiting during outbreaks believed, based on clinical criteria [22], to be due to norovirus, both in hospital wards (n = 24) as well as in the community (n = 4). Reproducibility was assessed by using a New Orleans 2009 sample. One ward outbreak sample containing the New Orleans 2009 strain was included in the first Illumina MiSeq run and two replicates on the second run. Two stool samples from symptomatic patients within ward outbreaks found to be qPCR negative for norovirus were also included on the MiSeq runs. For assembly comparison, sixteen samples collected from hospital ward outbreaks between 2009–2011 previously sequenced using Illumina HiSeq, together with four replicates (i.e. re-sequenced using the same total RNA) of these samples were included. These samples had been subjected to complete capillary sequencing, as described previously [13]. Quantitative polymerase chain reaction (qPCR) assays were performed for all samples using primers and Taqman probes as described [17].

#### Preparation and Illumina sequencing of RNA from faeces

Total RNA was extracted from faecal samples collected and prepared for sequencing using a modified RNA-Seq approach published recently [13]. Briefly, total RNA from faecal samples was isolated using the Fujifilm Quickgene DNA tissue kit SII (Fuji, Japan) under the manufacturer’s *RNA extraction from stool protocol*. Three hundred microlitres of supernatant from a 10% clarified emulsion was used as the lysate. One hundred nanograms of total RNA was extracted using a NEBNext mRNA sample preparation kit (NEB) and first strand synthesis performed using random primers as described, substituting Accuscript reverse transcriptase (Agilent), which displays higher fidelity than the enzyme used previously [23,24]. RNA-Seq library preparation was otherwise as described previously [13]. We multiplexed 16 samples per MiSeq run using custom index tags.

A total of 32 samples, including replicates were sequenced using two MiSeq runs, generating paired end reads of 150 nucleotide (nt) in length. The 20 samples (consisting of 16 samples and 4 replicates) previously sequenced using the Illumina HiSeq 2000, consisted of 100nt paired end reads.

### Bioinformatic processing

#### Norovirus reference sequences

477 full length norovirus genomes were downloaded from NCBI on 11 January 2013 (Additional file 1). We identified 91 sequence clusters with < =2% divergence over an alignment of at least 90% of the genome using BLASTClust, which is part of the NCBI Blast tools package. One representative genome was chosen from each cluster.

#### Identification of norovirus-like sequences among illumina reads

Illumina paired-end reads in which either or both sequences matched any cluster representative with a blastn e-value of <1×10^-8^ were retained. We refer to these sequences as Norovirus-like sequences (NLS). For each sample, the most similar reference sequence (MSRS) was identified on the basis of the number of matching read pairs.

#### Read processing

Reads were quality trimmed using the FastX Toolkit (version 0.0.13, Cold Spring Harbour Laboratory, New York, USA (http://hannonlab.cshl.edu/fastx_toolkit/)) with a Q-score cut-off of 15, adapters were removed using CutAdapt [25], and duplicate read-pairs were removed using custom Python scripts. We screened reads for library artefact sequences of the form 5′ ABA, where A and B are sequences from the same strand of a norovirus reference genome, A is a sequence of at least 18 nucleotides, and ABA is a short read of either 100 (for HiSeq runs) or 150nt (for MiSeq runs). The ABA pattern is not present in any of the canonical norovirus sequences and we found a low (<1/1000) frequency of such reads (Additional file 1). Singleton reads without matching partners following processing, were also removed.

#### De novo assembly

We compared four de-novo assembly algorithms, Velvet [18], Celera [19], Edena [20], and Vicuna [21], adapting their default settings as follows: in Velvet, k-mer and exp_cov were explored in a pairwise manner; for k-mer, values of 23 to 79 in steps of 4 were evaluated for the following values of exp_cov: auto, 1, 3, 10, 20, 30, 40, 50, 75, 100, 125, 150, 175, 200, 225, 250, 300, 400, 500, 600, 700, 800, 1000, 1200, 1500, 1800, 2000. Ins_length was specified at 250, based on known insert size distribution. For Edena, all read trimming lengths between 35 and 150 in steps of 5 were evaluated. In Vicuna, the following settings were used: minMSize = 9, maxOverhangSize = 2, Divergence = 15, Max read overhand = 4, Max contig overhang = 6, Seed kmer length = 9, Min contig overlap = 25, Min contig links =2, min identity = 90. In cases where multiple assembly parameters were compared, the setting(s) producing maximal N50 were selected. For all assemblers, contigs less than 300nt were removed from further analysis.

#### Assessing de novo assembly

To assess assembly quality and performance, we aligned each contig to its MSRS with blastn and a minimum e-value of 1×10^-8^. We computed the number of contigs, N50, percent of the MSRS which aligned to contigs, presence of gapped or multiple alignments to the MSRS.

Additionally, NLS reads were mapped to ether the Vicuna-derived contigs, or to contigs produced by capillary sequencing and contig assembly using the Staden package [26] and Geneious Pro software (version 6.1.6; Biomatters, New Zealand), using the built-in mapping algorithm using the ‘highest sensitivity’ setting. The results of the mapping, and the alignment of the contigs to the reference, were inspected manually.

#### Within-patient variant discovery

The NLS reads were mapped to the de-novo contig if a single contig was derived from the assembly. Mapping was performed with BOWTIE2 (version 2.1.0) [27] using the --very-sensitive flag. Samtools (version 0.1.19) [28] was used to covert alignments to pileup format. Pileup files were parsed to produce variant calls, excluding variants with base quality scores of less than 30, or mapping quality scores of less than 40. Additionally, we undertook a step designed to assess credibility of variants supported by low read numbers: we ignored variants for which, using an exact Binomial test (R 2.1.5, binom.test function), the proportion of reads supporting a variant relative to the reference was not significantly different (p > 0.01) than an expectation of 1×10^-4^. Finally, we only considered variants present at 1% or more of the reads.

#### Mis-assembly at the termini of contigs

In three samples, we observed that, although >99.5% coverage was obtained, two contigs were produced that differed at their termini. In each case, one contig had high (>500 reads) read support and represented part of a canonical norovirus genome, while the other was a rearrangement of a noroviral genome, with low (2 – 4 reads) support. These rearrangements disrupt open reading frames and are therefore unlikely to originate from a replication-competent virus (data not shown). In view of this, and the low read support for them, we manually removed the re-arranged, low-read terminus from these three samples and formed a single contig by manual editing.

#### Phylogenetic analyses and recombination detection

All unique genome sequences assembled from one or two contigs (n = 23) were aligned in Muscle v3.8 [29]. Only one of the three replicates has been included in our analyses. Maximum likelihood trees were constructed separately for ORF1 and ORF2/3 using PhyML 3.0 [30]. One hundred bootstrap replicates were performed for each tree.

We examined our dataset for the presence of recombinant sequences using the recombination detection software RDP3 [31]. We employed all 6 of the recombination detection methods available in RDP3’s automated scan, which provide the corresponding statistical significance of each event detected [31-36]. To identify as many potential breakpoints as possible, we included a genetically diverse set of 16 historical GII.4 reference sequences for which whole genome sequences were available from GenBank (accession numbers: FJ537134, X86557, AF145896, AY741811, EU310927, AB294779, AY502023, DQ369797, DQ078814, EF187497, EF684915, AB541319, GQ845368, HQ009513, GQ845367, JX459908).

#### Regulatory approval

The study was approved by Berkshire Research Ethics Committee on the 1st October 2010 (10/H0505/83) and information governance approval for processing of patient identifiable information was the UK National Information Governance Board (8-05(e)/2010).

#### Data deposition

The reads reported in this paper have been deposited in the European Nucleotide Archive Sequence Read Archive under study accession number PRJEB4318. The sequences produced by capillary sequencing used for validation have been similarly submitted under accession numbers HF952120-HF952135.

## Competing interests

The authors declare that they have no competing interests.

## Authors’ contributions

DHW conceived the study. THNW and DHW designed the study. THNW, BLD, JH, APG, DHW analysed the data and wrote the manuscript. THNW was involved with sample extraction and preparation. PP and AT were involved with sequencing the samples. JP, EGS, ES, JS, MHW were all involved with providing samples for this study and provided additional critique to this manuscript. KED, TEAP, DWC and DJW provided critique of the manuscript. All authors read and approved the manuscript.

## Supplementary Material

Additional file 1 **Norovirus genomes used as probes in analysis.** Spreadsheet displaying the original 477 full length norovirus genomes downloaded from NCBI on 11 January 2013 and used as probes for filtering to find Norovirus like sequences from sequenced reads. Heading abbreviations are as follows: Accession: the NCBI accession number; Description: the description of the virus; ShortDescription: a shorter description of the virus; AdditionDate: the date the sequence was added to NCBI; GI: The NCBI Gi; SeqLength: the length of the sequence. Click here for file
