# Supplementary material for: Whole genome sequencing and de novo assembly identifies Sydney-like variant noroviruses and recombinants during the winter 2012/2013 outbreak in England
Source: Virol J. 2013 Nov 13;10:335. doi: 10.1186/1743-422X-10-335 (PMC3874643; doi:10.1186/1743-422X-10-335)
Supplement: Additional file 1 — Norovirus genomes used as probes in analysis. Spreadsheet displaying the original 477 full length norovirus genomes downloaded from NCBI on 11 January 2013 and used as probes for filtering to find Norovirus like sequences from sequenced reads. Heading abbreviations are as follows: Accession: the NCBI accession number; Description: the description of the virus; ShortDescription: a shorter description of the virus; AdditionDate: the date the sequence was added to NCBI; GI: The NCBI Gi; SeqLength: the length of the sequence. [file 1743-422X-10-335-S1.pdf]

Accession	Description	ShortDescription	AdditionDate	GI	SeqLength
AF093797.1	Norwalk virus, complete genome.	Norwalk virus	29-SEP-2000	3769664	7598
AF504671.2	Norwalk virus strain Vietnam 026, complete genome.	Norwalk virus	16-FEB-2005	47175550	7540
M87661.2	Norwalk virus nonstructural polyprotein, 58 kd capsid protein, and orf3 genes, complete cds.	Norwalk virus	01-JUN-2006	106043086	7654
NC_001959.2	Norwalk virus, complete genome.	Norwalk virus	08-DEC-2008	106060735	7654
L07418.1	Southampton virus capsid protein, polyprotein, and ORF genes, complete cds.	Southampton virus	27-MAR-1996	1236787	7708
AY134748.1	Snow Mountain virus, complete genome.	Snow Mountain virus	01-JUL-2004	27762117	7537
X86557.1	Lordsdale virus complete genome.	Lordsdale virus	18-APR-2005	1008952	7555
AB039774.1	Norwalk-like virus genomic RNA, complete genome, isolate:SzUG1.	Norwalk-like virus	05-SEP-2002	22773784	7700
AB039775.1	Norwalk-like virus genomic RNA, complete genome, isolate:Saitama U1.	Norwalk-like virus	05-SEP-2002	22773788	7521
AB039776.1	Norwalk-like virus genomic RNA, complete genome, isolate:Saitama U3.	Norwalk-like virus	05-SEP-2002	22773792	7564
AB039777.1	Norwalk-like virus genomic RNA, complete genome, isolate:Saitama U4.	Norwalk-like virus	05-SEP-2002	22773796	7564
AB039778.1	Norwalk-like virus genomic RNA, complete genome, isolate:Saitama U16.	Norwalk-like virus	05-SEP-2002	22773800	7546
AB039779.1	Norwalk-like virus genomic RNA, complete genome, isolate:Saitama U17.	Norwalk-like virus	05-SEP-2002	22773804	7546
AB039780.1	Norwalk-like virus genomic RNA, complete genome, isolate:Saitama U25.	Norwalk-like virus	05-SEP-2002	22773808	7524
AB039781.1	Norwalk-like virus genomic RNA, complete genome, isolate:Saitama U18.	Norwalk-like virus	05-SEP-2002	22773812	7541
AB039782.1	Norwalk-like virus genomic RNA, complete genome, isolate:Saitama U201.	Norwalk-like virus	05-SEP-2002	22773816	7541
AB081723.1	Norwalk-like virus genomic RNA, complete genome, strain:WUG1.	Norwalk-like virus	04-SEP-2002	22773824	7688
AB045603.2	Norwalk-like virus genomic RNA, complete genome, specimen_voucher: Gifu'96.	Norwalk-like virus	10-JAN-2009	38229107	7521
AY126474.2	Norwalk-like virus isolate Bo/Dumfries/94/UK, complete genome.	Norwalk-like virus	05-SEP-2006	113951962	7311
AB083780.1	Norwalk-like virus genomic RNA, complete genome, strain: YURI.	Norwalk-like virus	10-JAN-2009	118722062	7560
AB083781.1	Norwalk-like virus genomic RNA, complete genome, strain: YURI 32073.	Norwalk-like virus	10-JAN-2009	118722066	7535
AB084071.1	Norwalk-like virus genomic RNA, complete genome, strain: GIFU'99.	Norwalk-like virus	10-JAN-2009	118722070	7562
AB126320.1	Norwalk-like virus genomic RNA, complete genome, strain: SW/NV/swine43/JP.	Norwalk-like virus	16-JAN-2009	118722074	7537
AB042808.1	Chiba virus genomic RNA, complete genome.	Chiba virus	06-JAN-2001	11275371	7697
AY772730.1	Norovirus Hu/NLV/GII/Neustrelitz260/2000/DE from Germany, complete genome.	Norovirus Hu/NLV/GII/Neustrelitz260/2000	01-NOV-2004	54694862	7579
AY032605.1	Human calicivirus Hu/NLV/GII/MD145-12/1987/US, complete genome.	Human calicivirus Hu/NLV/GII/MD145-12/1	29-JAN-2002	18025351	7556
AY228235.2	Murine norovirus 1 isolate Mu/NoV/GV/MNV1/2002/USA, complete genome.	Murine norovirus 1	20-JUN-2007	81174550	7382
DQ285629.1	Murine norovirus 1 clone CW1, complete genome.	Murine norovirus 1	20-JUN-2007	82754799	7382
NC_008311.1	Murine norovirus 1, complete genome.	Murine norovirus 1	04-JUN-2012	113478394	7382
EF014462.1	Murine norovirus 1 clone CW3, complete genome.	Murine norovirus 1	23-AUG-2007	116490118	7382
EU004654.1	Murine norovirus 1 clone CW2, complete genome.	Murine norovirus 1	19-SEP-2007	156186619	7382
EU004655.1	Murine norovirus 1 clone CW4, complete genome.	Murine norovirus 1	19-SEP-2007	156186623	7382
EU004656.1	Murine norovirus 1 clone CW5, complete genome.	Murine norovirus 1	19-SEP-2007	156186627	7382
EU004657.1	Murine norovirus 1 clone CW6, complete genome.	Murine norovirus 1	19-SEP-2007	156186631	7382

EU004658.1	Murine norovirus 1 clone CW7, complete genome.	Murine norovirus 1	19-SEP-2007	156186635	7382
EU004659.1	Murine norovirus 1 clone CW8, complete genome.	Murine norovirus 1	19-SEP-2007	156186639	7382
EU004660.1	Murine norovirus 1 clone CW9, complete genome.	Murine norovirus 1	19-SEP-2007	156186643	7382
EU004661.1	Murine norovirus 1 clone CW10, complete genome.	Murine norovirus 1	19-SEP-2007	156186647	7382
EU004662.1	Murine norovirus 1 clone CW11, complete genome.	Murine norovirus 1	19-SEP-2007	156186651	7382
AY237415.2	Human calicivirus strain Mc37, complete genome.	Human calicivirus strain Mc37	05-MAR-2004	44829133	7541
AY485642.1	Human calicivirus NLV/GII/Langen1061/2002/DE, complete genome.	Human calicivirus NLV/GII/Langen1061/20	22-DEC-2003	39980428	7558
AY581254.1	Human calicivirus Hu/NLV/Oxford/B5S22/2003/UK, complete genome.	Human calicivirus Hu/NLV/Oxford/B5S22/2	14-SEP-2004	46360469	7558
AY587983.1	Norovirus Hu/NLV/Oxford/B4S2/2002/UK, complete genome.	Norovirus Hu/NLV/Oxford/B4S2/2002/UK	14-SEP-2004	46519727	7558
AY587984.1	Norovirus Hu/NLV/Oxford/B4S5/2002/UK, complete genome.	Norovirus Hu/NLV/Oxford/B4S5/2002/UK	16-SEP-2004	46519731	7558
AY587985.1	Norovirus Hu/NLV/Oxford/B4S6/2002/UK, complete genome.	Norovirus Hu/NLV/Oxford/B4S6/2002/UK	16-SEP-2004	46519735	7558
AY587986.1	Norovirus Hu/NLV/Oxford/B4S4/2002/UK, complete genome.	Norovirus Hu/NLV/Oxford/B4S4/2002/UK	16-SEP-2004	46519739	7558
AY587987.1	Norovirus Hu/NLV/Oxford/B4S7/2002/UK, complete genome.	Norovirus Hu/NLV/Oxford/B4S7/2002/UK	16-SEP-2004	46519743	7558
AY587988.1	Norovirus Hu/NLV/Oxford/B4S1/2002/UK, complete genome.	Norovirus Hu/NLV/Oxford/B4S1/2002/UK	16-SEP-2004	46519747	7558
AY587989.1	Norovirus Hu/NLV/Oxford/B2S16/2002/UK, complete genome.	Norovirus Hu/NLV/Oxford/B2S16/2002/UK	16-SEP-2004	46519751	7558
AB187514.1	Norovirus Hu/GI/Otofuke/1979/JP genomic RNA, complete genome.	Norovirus Hu/GI/Otofuke/1979/JP	01-SEP-2005	74038598	7746
AY741811.1	Norovirus Hu/NLV/Dresden174/pUS-NorII/1997/GE, complete genome.	Norovirus Hu/NLV/Dresden174/pUS-NorII/	30-JUN-2006	53854911	7555
AY823305.2	Norovirus swine/GII/OH-QW125/03/US, complete genome.	Norovirus swine/GII/OH-QW125/03/US	27-JUN-2012	392973379	7612
DQ078814.2	Norovirus Hu/GII.4/Hunter504D/04O/AU, complete genome.	Norovirus Hu/GII.4/Hunter504D/04O/AU	14-SEP-2009	256916405	7559
AB220921.1	Norovirus Hu/Chiba/04-1050/2005/JP genomic RNA, complete genome.	Norovirus Hu/Chiba/04-1050/2005/JP	22-JUL-2006	110624497	7559
AF097917.5	Norovirus Bo/Newbury2/1976/UK, complete genome.	Norovirus Bo/Newbury2/1976/UK	19-JUN-2006	109254934	7311
FJ446720.1	Murine norovirus 3 strain K4, complete genome.	Murine norovirus 3	08-DEC-2009	216339278	7384
FJ446719.1	Murine norovirus 4 strain S18, complete genome.	Murine norovirus 4	08-DEC-2009	216339261	7383
DQ911368.1	Murine norovirus strain Berlin/04/06/DE, complete genome.	Murine norovirus	05-OCT-2006	115361555	7390
EF531290.1	Murine norovirus strain Berlin/05/06/DE, complete genome.	Murine norovirus	02-MAY-2007	145967359	7391
EF531291.1	Murine norovirus strain Berlin/06/06/DE, complete genome.	Murine norovirus	02-MAY-2007	145967363	7383
AB435514.1	Murine norovirus genomic RNA, complete genome, strain: S7-P2.	Murine norovirus	28-DEC-2010	219565720	7382
AB435515.1	Murine norovirus genomic RNA, complete genome, strain: S7-PP3.	Murine norovirus	10-JUN-2010	219565724	7382
HQ317203.1	Murine norovirus strain Guangzhou/K162/09/CHN, complete genome.	Murine norovirus	29-MAR-2011	326787489	7380
AB601769.1	Murine norovirus genomic RNA, complete genome, strain: MT30-2.	Murine norovirus	11-FEB-2012	375268440	7383
JX048594.1	Murine norovirus strain KHU-1, complete genome.	Murine norovirus	04-AUG-2012	398359617	7402
DQ369797.1	Norovirus Hu/Guangzhou/NVgz01/CHN, complete genome.	Norovirus Hu/Guangzhou/NVgz01/CHN	08-FEB-2006	86450249	7558
DQ456824.1	Norovirus Hu/MK04/2004/JP, complete genome.	Norovirus Hu/MK04/2004/JP	11-SEP-2012	91694050	7536
DQ658413.1	Norovirus Hu/GII.4/MD-2004/2004/US, complete genome.	Norovirus Hu/GII.4/MD-2004/2004/US	31-AUG-2007	110164874	7558
EF187497.2	Norovirus Hu/GII.4/Kenepuru/NZ327/2006/NZL, complete genome.	Norovirus Hu/GII.4/Kenepuru/NZ327/2006	06-FEB-2012	374674577	7559

EF650480.1	Murine norovirus 5 polyprotein gene, partial cds; and capsid protein gene, complete cds.	Murine norovirus 5	23-JUL-2007	152112995	6596
EF650481.1	Murine norovirus 6 polyprotein gene, partial cds; and capsid protein gene, complete cds.	Murine norovirus 6	23-JUL-2007	152112998	6610
EU004663.1	Murine norovirus GV/WU11/2005/USA, complete genome.	Murine norovirus GV/WU11/2005/USA	19-SEP-2007	156186655	7390
EU004664.1	Murine norovirus GV/WU12/2005/USA, complete genome.	Murine norovirus GV/WU12/2005/USA	19-SEP-2007	156186659	7384
EU004665.1	Murine norovirus GV/WU20/2005/USA, complete genome.	Murine norovirus GV/WU20/2005/USA	19-SEP-2007	156186663	7383
EU004666.1	Murine norovirus GV/WU21/2005/USA, complete genome.	Murine norovirus GV/WU21/2005/USA	19-SEP-2007	156186667	7380
EU004667.1	Murine norovirus GV/WU22/2005/USA, complete genome.	Murine norovirus GV/WU22/2005/USA	19-SEP-2007	156186671	7383
EU004668.1	Murine norovirus GV/WU23/2005/USA, complete genome.	Murine norovirus GV/WU23/2005/USA	19-SEP-2007	156186675	7383
EU004669.1	Murine norovirus GV/WU24/2005/USA, complete genome.	Murine norovirus GV/WU24/2005/USA	19-SEP-2007	156186679	7383
EU004670.1	Murine norovirus GV/WU25/2005/USA, complete genome.	Murine norovirus GV/WU25/2005/USA	19-SEP-2007	156186683	7383
EU004671.1	Murine norovirus GV/WU26/2005/USA, complete genome.	Murine norovirus GV/WU26/2005/USA	19-SEP-2007	156186687	7384
EU004672.1	Murine norovirus GV/CR1/2005/USA, complete genome.	Murine norovirus GV/CR1/2005/USA	19-SEP-2007	156186691	7382
EU004673.1	Murine norovirus GV/CR3/2005/USA, complete genome.	Murine norovirus GV/CR3/2005/USA	19-SEP-2007	156186695	7383
EU004674.1	Murine norovirus GV/CR4/2005/USA, complete genome.	Murine norovirus GV/CR4/2005/USA	19-SEP-2007	156186699	7382
EU004675.1	Murine norovirus GV/CR5/2005/USA, complete genome.	Murine norovirus GV/CR5/2005/USA	19-SEP-2007	156186703	7382
EU004676.1	Murine norovirus GV/CR6/2005/USA, complete genome.	Murine norovirus GV/CR6/2005/USA	19-SEP-2007	156186707	7383
JQ237823.1	Murine norovirus GV/CR6/2005/USA clone STL1, complete genome.	Murine norovirus GV/CR6/2005/USA	22-FEB-2012	374091607	7383
EU004677.1	Murine norovirus GV/CR7/2005/USA, complete genome.	Murine norovirus GV/CR7/2005/USA	19-SEP-2007	156186711	7380
EU004678.1	Murine norovirus GV/CR10/2005/USA, complete genome.	Murine norovirus GV/CR10/2005/USA	19-SEP-2007	156186715	7386
EU004679.1	Murine norovirus GV/CR11/2005/USA, complete genome.	Murine norovirus GV/CR11/2005/USA	19-SEP-2007	156186719	7386
EU004680.1	Murine norovirus GV/CR13/2005/USA, complete genome.	Murine norovirus GV/CR13/2005/USA	19-SEP-2007	156186723	7386
EU004681.1	Murine norovirus GV/CR15/2005/USA, complete genome.	Murine norovirus GV/CR15/2005/USA	19-SEP-2007	156186727	7389
EU004682.1	Murine norovirus GV/CR17/2005/USA, complete genome.	Murine norovirus GV/CR17/2005/USA	19-SEP-2007	156186731	7389
EU004683.1	Murine norovirus GV/CR18/2005/DEU, complete genome.	Murine norovirus GV/CR18/2005/DEU	19-SEP-2007	156186735	7381
EF684915.2	Norovirus Hu/GII.4/Shellharbour/NSW696T/2006/AUS, complete genome.	Norovirus Hu/GII.4/Shellharbour/NSW696	06-FEB-2012	374674581	7560
AB365435.1	Norovirus Hu/Texas/TCH04-577/2004/US genomic RNA, complete genome.	Norovirus Hu/Texas/TCH04-577/2004/US	25-APR-2008	186659484	7627
EU310927.1	Norovirus Hu/Houston/TCH186/2002/US, complete genome.	Norovirus Hu/Houston/TCH186/2002/US	24-DEC-2007	163637648	7559
AB447427.1	Norovirus Hu/GII-4/Hokkaido1/2006/JP genomic RNA, complete genome.	Norovirus Hu/GII-4/Hokkaido1/2006/JP	05-NOV-2008	198041339	7511
AB447428.1	Norovirus Hu/GII-4/Hokkaido2/2006/JP genomic RNA, complete genome.	Norovirus Hu/GII-4/Hokkaido2/2006/JP	05-NOV-2008	198041343	7511
AB447429.1	Norovirus Hu/GII-4/Hokkaido3/2006/JP genomic RNA, complete genome.	Norovirus Hu/GII-4/Hokkaido3/2006/JP	05-NOV-2008	198041347	7511
AB447430.1	Norovirus Hu/GII-4/Hokkaido4/2006/JP genomic RNA, complete genome.	Norovirus Hu/GII-4/Hokkaido4/2006/JP	05-NOV-2008	198041351	7511
AB447431.1	Norovirus Hu/GII-4/Hokkaido5/2006/JP genomic RNA, complete genome.	Norovirus Hu/GII-4/Hokkaido5/2006/JP	05-NOV-2008	198041355	7511
AB447432.1	Norovirus Hu/GII-4/Aomori1/2006/JP genomic RNA, complete genome.	Norovirus Hu/GII-4/Aomori1/2006/JP	05-NOV-2008	198041359	7502
AB447433.1	Norovirus Hu/GII-4/Aomori2/2006/JP genomic RNA, complete genome.	Norovirus Hu/GII-4/Aomori2/2006/JP	05-NOV-2008	198041363	7502
AB447434.1	Norovirus Hu/GII-4/Aomori4/2006/JP genomic RNA, complete genome.	Norovirus Hu/GII-4/Aomori4/2006/JP	05-NOV-2008	198041367	7511

AB447435.1	Norovirus Hu/GII-4/Aomori5/2006/JP genomic RNA, complete genome.	Norovirus Hu/GII-4/Aomori5/2006/JP	05-NOV-2008	198041371	7511
AB447436.1	Norovirus Hu/GII-4/Akita1/2006/JP genomic RNA, complete genome.	Norovirus Hu/GII-4/Akita1/2006/JP	05-NOV-2008	198041375	7511
AB447437.1	Norovirus Hu/GII-4/Akita2/2006/JP genomic RNA, complete genome.	Norovirus Hu/GII-4/Akita2/2006/JP	05-NOV-2008	198041379	7511
AB447438.1	Norovirus Hu/GII-4/Akita4/2006/JP genomic RNA, complete genome.	Norovirus Hu/GII-4/Akita4/2006/JP	05-NOV-2008	198041383	7511
AB447439.1	Norovirus Hu/GII-4/Akita5/2006/JP genomic RNA, complete genome.	Norovirus Hu/GII-4/Akita5/2006/JP	05-NOV-2008	198041387	7511
AB447440.1	Norovirus Hu/GII-4/Miyagi2/2006/JP genomic RNA, complete genome.	Norovirus Hu/GII-4/Miyagi2/2006/JP	05-NOV-2008	198041391	7511
AB447441.1	Norovirus Hu/GII-4/Miyagi4/2006/JP genomic RNA, complete genome.	Norovirus Hu/GII-4/Miyagi4/2006/JP	05-NOV-2008	198041395	7511
AB447442.1	Norovirus Hu/GII-4/Miyagi5/2006/JP genomic RNA, complete genome.	Norovirus Hu/GII-4/Miyagi5/2006/JP	05-NOV-2008	198041399	7511
AB447443.1	Norovirus Hu/GII-4/Toyama1/2006/JP genomic RNA, complete genome.	Norovirus Hu/GII-4/Toyama1/2006/JP	05-NOV-2008	198041403	7511
AB447444.1	Norovirus Hu/GII-4/Toyama4/2006/JP genomic RNA, complete genome.	Norovirus Hu/GII-4/Toyama4/2006/JP	05-NOV-2008	198041407	7511
AB447445.1	Norovirus Hu/GII-4/Toyama5/2006/JP genomic RNA, complete genome.	Norovirus Hu/GII-4/Toyama5/2006/JP	05-NOV-2008	198041411	7511
AB447446.1	Norovirus Hu/GII-4/Aichi3/2006/JP genomic RNA, complete genome.	Norovirus Hu/GII-4/Aichi3/2006/JP	05-NOV-2008	198041415	7511
AB447447.1	Norovirus Hu/GII-4/Aichi4/2006/JP genomic RNA, complete genome.	Norovirus Hu/GII-4/Aichi4/2006/JP	05-NOV-2008	198041419	7511
AB447448.1	Norovirus Hu/GII-4/Sakai2/2006/JP genomic RNA, complete genome.	Norovirus Hu/GII-4/Sakai2/2006/JP	05-NOV-2008	198041423	7511
AB447449.1	Norovirus Hu/GII-4/Sakai3/2006/JP genomic RNA, complete genome.	Norovirus Hu/GII-4/Sakai3/2006/JP	05-NOV-2008	198041427	7511
AB447450.1	Norovirus Hu/GII-4/Sakai4/2006/JP genomic RNA, complete genome.	Norovirus Hu/GII-4/Sakai4/2006/JP	05-NOV-2008	198041431	7511
AB447451.1	Norovirus Hu/GII-4/Hiroshima1/2006/JP genomic RNA, complete genome.	Norovirus Hu/GII-4/Hiroshima1/2006/JP	05-NOV-2008	198041435	7511
AB447452.1	Norovirus Hu/GII-4/Hiroshima2/2006/JP genomic RNA, complete genome.	Norovirus Hu/GII-4/Hiroshima2/2006/JP	05-NOV-2008	198041439	7511
AB447453.1	Norovirus Hu/GII-4/Ehime1/2006/JP genomic RNA, complete genome.	Norovirus Hu/GII-4/Ehime1/2006/JP	05-NOV-2008	198041443	7511
AB447454.1	Norovirus Hu/GII-4/Ehime2/2006/JP genomic RNA, complete genome.	Norovirus Hu/GII-4/Ehime2/2006/JP	05-NOV-2008	198041447	7511
AB447455.1	Norovirus Hu/GII-4/Ehime5/2006/JP genomic RNA, complete genome.	Norovirus Hu/GII-4/Ehime5/2006/JP	05-NOV-2008	198041451	7511
AB447456.1	Norovirus Hu/GII-4/Saga1/2006/JP genomic RNA, complete genome.	Norovirus Hu/GII-4/Saga1/2006/JP	05-NOV-2008	198041455	7511
AB447457.1	Norovirus Hu/GII-4/Saga4/2006/JP genomic RNA, complete genome.	Norovirus Hu/GII-4/Saga4/2006/JP	05-NOV-2008	198041459	7511
AB447458.1	Norovirus Hu/GII-4/Saga5/2006/JP genomic RNA, complete genome.	Norovirus Hu/GII-4/Saga5/2006/JP	05-NOV-2008	198041463	7511
AB447459.1	Norovirus Hu/GII-4/Kumamoto1/2006/JP genomic RNA, complete genome.	Norovirus Hu/GII-4/Kumamoto1/2006/JP	05-NOV-2008	198041467	7511
AB447460.1	Norovirus Hu/GII-4/Kumamoto2/2006/JP genomic RNA, complete genome.	Norovirus Hu/GII-4/Kumamoto2/2006/JP	05-NOV-2008	198041471	7511
AB447461.1	Norovirus Hu/GII-4/Kumamoto3/2006/JP genomic RNA, complete genome.	Norovirus Hu/GII-4/Kumamoto3/2006/JP	05-NOV-2008	198041475	7511
AB447462.1	Norovirus Hu/GII-4/Kumamoto4/2006/JP genomic RNA, complete genome.	Norovirus Hu/GII-4/Kumamoto4/2006/JP	05-NOV-2008	198041479	7511
AB447463.1	Norovirus Hu/GII-4/Kumamoto5/2006/JP genomic RNA, complete genome.	Norovirus Hu/GII-4/Kumamoto5/2006/JP	05-NOV-2008	198041483	7511
EU854589.1	Norovirus mouse/Hannover1/2007/DEU, complete genome.	Norovirus mouse/Hannover1/2007/DEU	01-AUG-2008	194462546	7383
EU794907.1	Norovirus Bo/GIII/B309/2003/BEL polyprotein, capsid, and VP2 genes, complete cds.	Norovirus Bo/GIII/B309/2003/BEL	23-OCT-2012	209980410	7317
EU921344.2	Norovirus Hu/Pune/PC15/2006/India, complete genome.	Norovirus Hu/Pune/PC15/2006/India	03-AUG-2010	302138819	7559
EU921388.2	Norovirus Hu/Pune/PC51/2007/India, complete genome.	Norovirus Hu/Pune/PC51/2007/India	03-AUG-2010	302138823	7559
EU921389.2	Norovirus Hu/Pune/PC52/2007/India, complete genome.	Norovirus Hu/Pune/PC52/2007/India	03-AUG-2010	302138827	7547
FJ514242.1	Norovirus Hu/GII-4/CUK-3/2008/KR, complete genome.	Norovirus Hu/GII-4/CUK-3/2008/KR	18-APR-2011	219810312	7559

FJ515294.1	Norovirus Hu/GI.2/Leuven/2003/BEL, complete genome.	Norovirus Hu/GI.2/Leuven/2003/BEL	01-JAN-2010	256013120	7699
FJ537135.1	Norovirus Hu/GII.4/CHDC2094/1974/US, complete genome.	Norovirus Hu/GII.4/CHDC2094/1974/US	02-NOV-2009	254654019	7576
FJ537136.1	Norovirus Hu/GII.4/CHDC3967/1988/US, complete genome.	Norovirus Hu/GII.4/CHDC3967/1988/US	02-NOV-2009	254654023	7580
FJ537137.1	Norovirus Hu/GII.4/CHDC4108/1987/US, complete genome.	Norovirus Hu/GII.4/CHDC4108/1987/US	02-NOV-2009	254654027	7580
FJ537138.1	Norovirus Hu/GII.4/CHDC4871/1977/US, complete genome.	Norovirus Hu/GII.4/CHDC4871/1977/US	02-NOV-2009	254654031	7580
FJ537134.1	Norovirus Hu/GII.4/CHDC5191/1974/US, complete genome.	Norovirus Hu/GII.4/CHDC5191/1974/US	02-NOV-2009	254654015	7580
FJ692500.1	Norovirus dog/GVI.1/HKU_Ca026F/2007/HKG ORF1 polyprotein, VP1 capsid protein, and VP2 minor stru	Norovirus dog/GVI.1/HKU_Ca026F/2007/H	18-AUG-2012	386688651	7637
FJ692501.1	Norovirus dog/GVI.1/HKU_Ca035F/2007/HKG ORF1 polyprotein, VP1 capsid protein, and VP2 minor stru	Norovirus dog/GVI.1/HKU_Ca035F/2007/H	18-AUG-2012	386688653	7637
GQ845024.2	Norovirus Hu/GII.4/Rathmines/NSW287R/2007/AUS, complete genome.	Norovirus Hu/GII.4/Rathmines/NSW287R/	06-FEB-2012	374674584	7562
GQ845367.2	Norovirus Hu/GII.4/Orange/NSW001P/2008/AU, complete genome.	Norovirus Hu/GII.4/Orange/NSW001P/200	02-DEC-2010	311977260	7559
GQ845370.2	Norovirus Hu/GII.g-GII.12/StGeorge/NSW199U/2008/AU, complete genome.	Norovirus Hu/GII.g-GII.12/StGeorge/NSW	08-NOV-2011	311788785	7523
GQ845366.2	Norovirus Hu/GII.4/Westmead/NSW3639/2008/AUS, complete genome.	Norovirus Hu/GII.4/Westmead/NSW3639/	06-FEB-2012	374674587	7560
GQ845369.3	Norovirus Hu/GII.4/Armidale/NSW390I/2008/AU, complete genome.	Norovirus Hu/GII.4/Armidale/NSW390I/20	06-FEB-2012	374674593	7560
GQ845368.2	Norovirus Hu/GII.4/Sutherland/NSW505G/2007/AUS, complete genome.	Norovirus Hu/GII.4/Sutherland/NSW505G/	06-FEB-2012	374674590	7559
GU017903.2	Norovirus Hu/8533/Maizuru/08/JPN, complete genome.	Norovirus Hu/8533/Maizuru/08/JPN	03-FEB-2010	285118921	7529
GU325839.2	Norovirus Hu/GII.4/HS194/2009/US, complete genome.	Norovirus Hu/GII.4/HS194/2009/US	06-SEP-2011	334178596	7560
AB541190.1	Norovirus Hu/GII-4/2007a_ORF2-3/JP genes for polyprotein, VP1, VP2, partial and complete cds.	Norovirus Hu/GII-4/2007a_ORF2-3/JP	03-AUG-2010	302128177	2751
AB541192.1	Norovirus Hu/GII-4/2007b_ORF2-3/JP genes for polyprotein, VP1, VP2, partial and complete cds.	Norovirus Hu/GII-4/2007b_ORF2-3/JP	03-AUG-2010	302128184	2748
AB541195.1	Norovirus Hu/GII-4/2008a_ORF2-3/JP genes for polyprotein, VP1, VP2, partial and complete cds.	Norovirus Hu/GII-4/2008a_ORF2-3/JP	03-AUG-2010	302128194	2751
AB541198.1	Norovirus Hu/GII-4/2008b_ORF2-3/JP genes for polyprotein, VP1, VP2, partial and complete cds.	Norovirus Hu/GII-4/2008b_ORF2-3/JP	03-AUG-2010	302128204	2751
AB541201.1	Norovirus Hu/GII-4/Aichi1/2007/JP genomic RNA, complete genome.	Norovirus Hu/GII-4/Aichi1/2007/JP	03-AUG-2010	302128214	7509
AB541202.1	Norovirus Hu/GII-4/Aichi1/2008/JP genomic RNA, complete genome.	Norovirus Hu/GII-4/Aichi1/2008/JP	03-AUG-2010	302128218	7509
AB541203.1	Norovirus Hu/GII-4/Aichi2/2007/JP genomic RNA, complete genome.	Norovirus Hu/GII-4/Aichi2/2007/JP	03-AUG-2010	302128222	7509
AB541204.1	Norovirus Hu/GII-4/Aichi2/2008/JP genomic RNA, complete genome.	Norovirus Hu/GII-4/Aichi2/2008/JP	03-AUG-2010	302128226	7509
AB541205.1	Norovirus Hu/GII-4/Aichi3/2007/JP genomic RNA, complete genome.	Norovirus Hu/GII-4/Aichi3/2007/JP	03-AUG-2010	302128230	7509
AB541206.1	Norovirus Hu/GII-4/Aichi3/2008/JP genomic RNA, complete genome.	Norovirus Hu/GII-4/Aichi3/2008/JP	03-AUG-2010	302128234	7509
AB541207.1	Norovirus Hu/GII-4/Aichi4/2007/JP genomic RNA, complete genome.	Norovirus Hu/GII-4/Aichi4/2007/JP	03-AUG-2010	302128238	7509
AB541208.1	Norovirus Hu/GII-4/Aichi4/2008/JP genomic RNA, complete genome.	Norovirus Hu/GII-4/Aichi4/2008/JP	03-AUG-2010	302128242	7509
AB541209.1	Norovirus Hu/GII-4/Aichi5/2007/JP genomic RNA, complete genome.	Norovirus Hu/GII-4/Aichi5/2007/JP	03-AUG-2010	302128246	7509
AB541210.1	Norovirus Hu/GII-4/Aichi5/2008/JP genomic RNA, complete genome.	Norovirus Hu/GII-4/Aichi5/2008/JP	03-AUG-2010	302128250	7509
AB541211.1	Norovirus Hu/GII-4/Akita1/2007/JP genomic RNA, complete genome.	Norovirus Hu/GII-4/Akita1/2007/JP	03-AUG-2010	302128254	7509
AB541212.1	Norovirus Hu/GII-4/Akita1/2008/JP genomic RNA, nearly complete genome.	Norovirus Hu/GII-4/Akita1/2008/JP	03-AUG-2010	302128258	7404
AB541213.1	Norovirus Hu/GII-4/Akita2/2008/JP genomic RNA, complete genome.	Norovirus Hu/GII-4/Akita2/2008/JP	03-AUG-2010	302128262	7509
AB541214.1	Norovirus Hu/GII-4/Akita3/2007/JP genomic RNA, complete genome.	Norovirus Hu/GII-4/Akita3/2007/JP	03-AUG-2010	302128266	7509
AB541215.1	Norovirus Hu/GII-4/Akita3/2008/JP genomic RNA, complete genome.	Norovirus Hu/GII-4/Akita3/2008/JP	03-AUG-2010	302128270	7509

AB541216.1	Norovirus Hu/GII-4/Akita4/2008/JP genomic RNA, complete genome.	Norovirus Hu/GII-4/Akita4/2008/JP	03-AUG-2010	302128274	7509
AB541217.1	Norovirus Hu/GII-4/Akita5/2007/JP genomic RNA, complete genome.	Norovirus Hu/GII-4/Akita5/2007/JP	03-AUG-2010	302128278	7509
AB541218.1	Norovirus Hu/GII-4/Aomori1/2007/JP genomic RNA, complete genome.	Norovirus Hu/GII-4/Aomori1/2007/JP	03-AUG-2010	302128282	7509
AB541219.1	Norovirus Hu/GII-4/Aomori1/2008/JP genomic RNA, complete genome.	Norovirus Hu/GII-4/Aomori1/2008/JP	03-AUG-2010	302128286	7509
AB541220.1	Norovirus Hu/GII-4/Aomori2/2007/JP genomic RNA, complete genome.	Norovirus Hu/GII-4/Aomori2/2007/JP	03-AUG-2010	302128290	7509
AB541221.1	Norovirus Hu/GII-4/Aomori2/2008/JP genomic RNA, nearly complete genome.	Norovirus Hu/GII-4/Aomori2/2008/JP	03-AUG-2010	302128294	7404
AB541222.1	Norovirus Hu/GII-4/Aomori3/2007/JP genomic RNA, complete genome.	Norovirus Hu/GII-4/Aomori3/2007/JP	03-AUG-2010	302128298	7509
AB541223.1	Norovirus Hu/GII-4/Aomori3/2008/JP genomic RNA, complete genome.	Norovirus Hu/GII-4/Aomori3/2008/JP	03-AUG-2010	302128302	7509
AB541224.1	Norovirus Hu/GII-4/Aomori4/2007/JP genomic RNA, complete genome.	Norovirus Hu/GII-4/Aomori4/2007/JP	03-AUG-2010	302128306	7509
AB541225.1	Norovirus Hu/GII-4/Aomori4/2008/JP genomic RNA, complete genome.	Norovirus Hu/GII-4/Aomori4/2008/JP	03-AUG-2010	302128310	7509
AB541226.1	Norovirus Hu/GII-4/Aomori5/2007/JP genomic RNA, complete genome.	Norovirus Hu/GII-4/Aomori5/2007/JP	03-AUG-2010	302128314	7509
AB541227.1	Norovirus Hu/GII-4/Aomori5/2008/JP genomic RNA, complete genome.	Norovirus Hu/GII-4/Aomori5/2008/JP	03-AUG-2010	302128318	7509
AB541228.1	Norovirus Hu/GII-4/Chiba1/2007/JP genomic RNA, complete genome.	Norovirus Hu/GII-4/Chiba1/2007/JP	03-AUG-2010	302128322	7509
AB541229.1	Norovirus Hu/GII-4/Chiba1/2008/JP genomic RNA, complete genome.	Norovirus Hu/GII-4/Chiba1/2008/JP	03-AUG-2010	302128326	7509
AB541230.1	Norovirus Hu/GII-4/Chiba2/2007/JP genomic RNA, complete genome.	Norovirus Hu/GII-4/Chiba2/2007/JP	03-AUG-2010	302128330	7509
AB541231.1	Norovirus Hu/GII-4/Chiba2/2008/JP genomic RNA, complete genome.	Norovirus Hu/GII-4/Chiba2/2008/JP	03-AUG-2010	302128334	7509
AB541232.1	Norovirus Hu/GII-4/Chiba4/2007/JP genomic RNA, complete genome.	Norovirus Hu/GII-4/Chiba4/2007/JP	03-AUG-2010	302128338	7509
AB541233.1	Norovirus Hu/GII-4/Chiba4/2008/JP genomic RNA, complete genome.	Norovirus Hu/GII-4/Chiba4/2008/JP	03-AUG-2010	302128342	7497
AB541234.1	Norovirus Hu/GII-4/Chiba5/2007/JP genomic RNA, complete genome.	Norovirus Hu/GII-4/Chiba5/2007/JP	03-AUG-2010	302128346	7509
AB541235.1	Norovirus Hu/GII-4/Chiba5/2008/JP genomic RNA, complete genome.	Norovirus Hu/GII-4/Chiba5/2008/JP	03-AUG-2010	302128350	7509
AB541236.1	Norovirus Hu/GII-4/Ehime1/2007/JP genomic RNA, complete genome.	Norovirus Hu/GII-4/Ehime1/2007/JP	03-AUG-2010	302128354	7509
AB541237.1	Norovirus Hu/GII-4/Ehime1/2008/JP genomic RNA, complete genome.	Norovirus Hu/GII-4/Ehime1/2008/JP	03-AUG-2010	302128358	7509
AB541238.1	Norovirus Hu/GII-4/Ehime2/2007/JP genomic RNA, complete genome.	Norovirus Hu/GII-4/Ehime2/2007/JP	03-AUG-2010	302128362	7509
AB541239.1	Norovirus Hu/GII-4/Ehime3/2007/JP genomic RNA, complete genome.	Norovirus Hu/GII-4/Ehime3/2007/JP	03-AUG-2010	302128366	7509
AB541240.1	Norovirus Hu/GII-4/Ehime3/2008/JP genomic RNA, complete genome.	Norovirus Hu/GII-4/Ehime3/2008/JP	03-AUG-2010	302128370	7509
AB541241.1	Norovirus Hu/GII-4/Ehime4/2007/JP genomic RNA, complete genome.	Norovirus Hu/GII-4/Ehime4/2007/JP	03-AUG-2010	302128374	7509
AB541242.1	Norovirus Hu/GII-4/Ehime4/2008/JP genomic RNA, complete genome.	Norovirus Hu/GII-4/Ehime4/2008/JP	03-AUG-2010	302128378	7509
AB541243.1	Norovirus Hu/GII-4/Ehime5/2008/JP genomic RNA, complete genome.	Norovirus Hu/GII-4/Ehime5/2008/JP	03-AUG-2010	302128382	7509
AB541244.1	Norovirus Hu/GII-4/Fukui1/2008/JP genomic RNA, complete genome.	Norovirus Hu/GII-4/Fukui1/2008/JP	03-AUG-2010	302128386	7509
AB541245.1	Norovirus Hu/GII-4/Fukui2/2007/JP genomic RNA, complete genome.	Norovirus Hu/GII-4/Fukui2/2007/JP	03-AUG-2010	302128390	7509
AB541246.1	Norovirus Hu/GII-4/Fukui2/2008/JP genomic RNA, complete genome.	Norovirus Hu/GII-4/Fukui2/2008/JP	03-AUG-2010	302128394	7509
AB541247.1	Norovirus Hu/GII-4/Fukui4/2007/JP genomic RNA, complete genome.	Norovirus Hu/GII-4/Fukui4/2007/JP	03-AUG-2010	302128398	7509
AB541248.1	Norovirus Hu/GII-4/Fukui4/2008/JP genomic RNA, complete genome.	Norovirus Hu/GII-4/Fukui4/2008/JP	03-AUG-2010	302128402	7509
AB541249.1	Norovirus Hu/GII-4/Fukui5/2007/JP genomic RNA, complete genome.	Norovirus Hu/GII-4/Fukui5/2007/JP	03-AUG-2010	302128406	7509
AB541250.1	Norovirus Hu/GII-4/Fukui5/2008/JP genomic RNA, complete genome.	Norovirus Hu/GII-4/Fukui5/2008/JP	03-AUG-2010	302128410	7509

AB541251.1	Norovirus Hu/GII-4/Hiroshima1/2007/JP genomic RNA, complete genome.	Norovirus Hu/GII-4/Hiroshima1/2007/JP	03-AUG-2010	302128414	7509
AB541252.1	Norovirus Hu/GII-4/Hiroshima1/2008/JP genomic RNA, complete genome.	Norovirus Hu/GII-4/Hiroshima1/2008/JP	03-AUG-2010	302128418	7509
AB541253.1	Norovirus Hu/GII-4/Hiroshima2/2007/JP genomic RNA, complete genome.	Norovirus Hu/GII-4/Hiroshima2/2007/JP	03-AUG-2010	302128422	7509
AB541254.1	Norovirus Hu/GII-4/Hiroshima2/2008/JP genomic RNA, complete genome.	Norovirus Hu/GII-4/Hiroshima2/2008/JP	03-AUG-2010	302128426	7509
AB541255.1	Norovirus Hu/GII-4/Hiroshima3/2007/JP genomic RNA, complete genome.	Norovirus Hu/GII-4/Hiroshima3/2007/JP	03-AUG-2010	302128430	7509
AB541256.1	Norovirus Hu/GII-4/Hiroshima3/2008/JP genomic RNA, complete genome.	Norovirus Hu/GII-4/Hiroshima3/2008/JP	03-AUG-2010	302128434	7509
AB541257.1	Norovirus Hu/GII-4/Hiroshima4/2007/JP genomic RNA, complete genome.	Norovirus Hu/GII-4/Hiroshima4/2007/JP	03-AUG-2010	302128438	7509
AB541258.1	Norovirus Hu/GII-4/Hiroshima4/2008/JP genomic RNA, complete genome.	Norovirus Hu/GII-4/Hiroshima4/2008/JP	03-AUG-2010	302128442	7509
AB541259.1	Norovirus Hu/GII-4/Hiroshima5/2008/JP genomic RNA, complete genome.	Norovirus Hu/GII-4/Hiroshima5/2008/JP	03-AUG-2010	302128446	7509
AB541260.1	Norovirus Hu/GII-4/Hokkaido1/2007/JP genomic RNA, complete genome.	Norovirus Hu/GII-4/Hokkaido1/2007/JP	03-AUG-2010	302128450	7509
AB541261.1	Norovirus Hu/GII-4/Hokkaido1/2008/JP genomic RNA, complete genome.	Norovirus Hu/GII-4/Hokkaido1/2008/JP	03-AUG-2010	302128454	7509
AB541262.1	Norovirus Hu/GII-4/Hokkaido2/2007/JP genomic RNA, complete genome.	Norovirus Hu/GII-4/Hokkaido2/2007/JP	03-AUG-2010	302128458	7509
AB541263.1	Norovirus Hu/GII-4/Hokkaido2/2008/JP genomic RNA, complete genome.	Norovirus Hu/GII-4/Hokkaido2/2008/JP	03-AUG-2010	302128462	7509
AB541264.1	Norovirus Hu/GII-4/Hokkaido3/2008/JP genomic RNA, complete genome.	Norovirus Hu/GII-4/Hokkaido3/2008/JP	03-AUG-2010	302128466	7509
AB541265.1	Norovirus Hu/GII-4/Hokkaido4/2007/JP genomic RNA, complete genome.	Norovirus Hu/GII-4/Hokkaido4/2007/JP	03-AUG-2010	302128470	7509
AB541266.1	Norovirus Hu/GII-4/Hokkaido4/2008/JP genomic RNA, complete genome.	Norovirus Hu/GII-4/Hokkaido4/2008/JP	03-AUG-2010	302128474	7509
AB541267.1	Norovirus Hu/GII-4/Hokkaido5/2007/JP genomic RNA, complete genome.	Norovirus Hu/GII-4/Hokkaido5/2007/JP	03-AUG-2010	302128478	7509
AB541268.1	Norovirus Hu/GII-4/Hokkaido5/2008/JP genomic RNA, complete genome.	Norovirus Hu/GII-4/Hokkaido5/2008/JP	03-AUG-2010	302128482	7509
AB541269.1	Norovirus Hu/GII-4/Iwate1/2008/JP genomic RNA, complete genome.	Norovirus Hu/GII-4/Iwate1/2008/JP	03-AUG-2010	302128486	7509
AB541270.1	Norovirus Hu/GII-4/Iwate2/2007/JP genomic RNA, complete genome.	Norovirus Hu/GII-4/Iwate2/2007/JP	03-AUG-2010	302128490	7509
AB541271.1	Norovirus Hu/GII-4/Iwate3/2007/JP genomic RNA, complete genome.	Norovirus Hu/GII-4/Iwate3/2007/JP	03-AUG-2010	302128494	7509
AB541272.1	Norovirus Hu/GII-4/Iwate3/2008/JP genomic RNA, complete genome.	Norovirus Hu/GII-4/Iwate3/2008/JP	03-AUG-2010	302128498	7509
AB541273.1	Norovirus Hu/GII-4/Iwate4/2007/JP genomic RNA, complete genome.	Norovirus Hu/GII-4/Iwate4/2007/JP	03-AUG-2010	302128502	7509
AB541274.1	Norovirus Hu/GII-4/Iwate4/2008/JP genomic RNA, complete genome.	Norovirus Hu/GII-4/Iwate4/2008/JP	03-AUG-2010	302128506	7509
AB541275.1	Norovirus Hu/GII-4/Iwate5/2007/JP genomic RNA, complete genome.	Norovirus Hu/GII-4/Iwate5/2007/JP	03-AUG-2010	302128510	7506
AB541276.1	Norovirus Hu/GII-4/Iwate5/2008/JP genomic RNA, complete genome.	Norovirus Hu/GII-4/Iwate5/2008/JP	03-AUG-2010	302128514	7509
AB541277.1	Norovirus Hu/GII-4/Kumamoto1/2007/JP genomic RNA, complete genome.	Norovirus Hu/GII-4/Kumamoto1/2007/JP	03-AUG-2010	302128518	7509
AB541278.1	Norovirus Hu/GII-4/Kumamoto2/2007/JP genomic RNA, complete genome.	Norovirus Hu/GII-4/Kumamoto2/2007/JP	03-AUG-2010	302128522	7509
AB541279.1	Norovirus Hu/GII-4/Kumamoto3/2007/JP genomic RNA, complete genome.	Norovirus Hu/GII-4/Kumamoto3/2007/JP	03-AUG-2010	302128526	7509
AB541280.1	Norovirus Hu/GII-4/Kumamoto4/2007/JP genomic RNA, complete genome.	Norovirus Hu/GII-4/Kumamoto4/2007/JP	03-AUG-2010	302128530	7509
AB541281.1	Norovirus Hu/GII-4/Miyagi1/2007/JP genomic RNA, complete genome.	Norovirus Hu/GII-4/Miyagi1/2007/JP	03-AUG-2010	302128534	7509
AB541282.1	Norovirus Hu/GII-4/Miyagi2/2007/JP genomic RNA, complete genome.	Norovirus Hu/GII-4/Miyagi2/2007/JP	03-AUG-2010	302128538	7509
AB541283.1	Norovirus Hu/GII-4/Miyagi3/2007/JP genomic RNA, complete genome.	Norovirus Hu/GII-4/Miyagi3/2007/JP	03-AUG-2010	302128542	7509
AB541284.1	Norovirus Hu/GII-4/Miyagi5/2008/JP genomic RNA, complete genome.	Norovirus Hu/GII-4/Miyagi5/2008/JP	03-AUG-2010	302128546	7509
AB541285.1	Norovirus Hu/GII-4/Miyazaki10/2008/JP genomic RNA, complete genome.	Norovirus Hu/GII-4/Miyazaki10/2008/JP	03-AUG-2010	302128550	7509

AB541286.1	Norovirus Hu/GII-4/Miyazaki12/2008/JP genomic RNA, complete genome.	Norovirus Hu/GII-4/Miyazaki12/2008/JP	03-AUG-2010	302128554	7509
AB541287.1	Norovirus Hu/GII-4/Miyazaki13/2008/JP genomic RNA, complete genome.	Norovirus Hu/GII-4/Miyazaki13/2008/JP	03-AUG-2010	302128558	7509
AB541288.1	Norovirus Hu/GII-4/Miyazaki1/2007/JP genomic RNA, complete genome.	Norovirus Hu/GII-4/Miyazaki1/2007/JP	03-AUG-2010	302128562	7509
AB541289.1	Norovirus Hu/GII-4/Miyazaki1/2008/JP genomic RNA, complete genome.	Norovirus Hu/GII-4/Miyazaki1/2008/JP	03-AUG-2010	302128566	7509
AB541290.1	Norovirus Hu/GII-4/Miyazaki2/2007/JP genomic RNA, complete genome.	Norovirus Hu/GII-4/Miyazaki2/2007/JP	03-AUG-2010	302128570	7509
AB541291.1	Norovirus Hu/GII-4/Miyazaki2/2008/JP genomic RNA, complete genome.	Norovirus Hu/GII-4/Miyazaki2/2008/JP	03-AUG-2010	302128574	7509
AB541292.1	Norovirus Hu/GII-4/Miyazaki3/2007/JP genomic RNA, complete genome.	Norovirus Hu/GII-4/Miyazaki3/2007/JP	03-AUG-2010	302128578	7509
AB541293.1	Norovirus Hu/GII-4/Miyazaki3/2008/JP genomic RNA, complete genome.	Norovirus Hu/GII-4/Miyazaki3/2008/JP	03-AUG-2010	302128582	7509
AB541294.1	Norovirus Hu/GII-4/Miyazaki4/2007/JP genomic RNA, complete genome.	Norovirus Hu/GII-4/Miyazaki4/2007/JP	03-AUG-2010	302128586	7509
AB541295.1	Norovirus Hu/GII-4/Miyazaki4/2008/JP genomic RNA, complete genome.	Norovirus Hu/GII-4/Miyazaki4/2008/JP	03-AUG-2010	302128590	7509
AB541296.1	Norovirus Hu/GII-4/Miyazaki5/2007/JP genomic RNA, complete genome.	Norovirus Hu/GII-4/Miyazaki5/2007/JP	03-AUG-2010	302128594	7509
AB541297.1	Norovirus Hu/GII-4/Miyazaki6/2008/JP genomic RNA, complete genome.	Norovirus Hu/GII-4/Miyazaki6/2008/JP	03-AUG-2010	302128598	7509
AB541298.1	Norovirus Hu/GII-4/Miyazaki7/2008/JP genomic RNA, complete genome.	Norovirus Hu/GII-4/Miyazaki7/2008/JP	03-AUG-2010	302128602	7509
AB541299.1	Norovirus Hu/GII-4/Miyazaki8/2008/JP genomic RNA, complete genome.	Norovirus Hu/GII-4/Miyazaki8/2008/JP	03-AUG-2010	302128606	7509
AB541300.1	Norovirus Hu/GII-4/Miyazaki9/2008/JP genomic RNA, nearly complete genome.	Norovirus Hu/GII-4/Miyazaki9/2008/JP	03-AUG-2010	302128610	7263
AB541301.1	Norovirus Hu/GII-4/Nagano1/2007/JP genomic RNA, complete genome.	Norovirus Hu/GII-4/Nagano1/2007/JP	03-AUG-2010	302128614	7509
AB541302.1	Norovirus Hu/GII-4/Nagano1/2008/JP genomic RNA, complete genome.	Norovirus Hu/GII-4/Nagano1/2008/JP	03-AUG-2010	302128618	7509
AB541303.1	Norovirus Hu/GII-4/Nagano2/2007/JP genomic RNA, complete genome.	Norovirus Hu/GII-4/Nagano2/2007/JP	03-AUG-2010	302128622	7509
AB541304.1	Norovirus Hu/GII-4/Nagano2/2008/JP genomic RNA, complete genome.	Norovirus Hu/GII-4/Nagano2/2008/JP	03-AUG-2010	302128626	7509
AB541305.1	Norovirus Hu/GII-4/Nagano3/2007/JP genomic RNA, complete genome.	Norovirus Hu/GII-4/Nagano3/2007/JP	03-AUG-2010	302128630	7512
AB541306.1	Norovirus Hu/GII-4/Nagano3/2008/JP genomic RNA, complete genome.	Norovirus Hu/GII-4/Nagano3/2008/JP	03-AUG-2010	302128634	7509
AB541307.1	Norovirus Hu/GII-4/Nagano4/2008/JP genomic RNA, complete genome.	Norovirus Hu/GII-4/Nagano4/2008/JP	03-AUG-2010	302128638	7509
AB541308.1	Norovirus Hu/GII-4/Nagano5/2007/JP genomic RNA, complete genome.	Norovirus Hu/GII-4/Nagano5/2007/JP	03-AUG-2010	302128642	7509
AB541309.1	Norovirus Hu/GII-4/Niigata1/2007/JP genomic RNA, complete genome.	Norovirus Hu/GII-4/Niigata1/2007/JP	03-AUG-2010	302128646	7509
AB541310.1	Norovirus Hu/GII-4/Niigata1/2008/JP genomic RNA, complete genome.	Norovirus Hu/GII-4/Niigata1/2008/JP	03-AUG-2010	302128650	7509
AB541311.1	Norovirus Hu/GII-4/Niigata2/2007/JP genomic RNA, complete genome.	Norovirus Hu/GII-4/Niigata2/2007/JP	03-AUG-2010	302128654	7509
AB541312.1	Norovirus Hu/GII-4/Niigata2/2008/JP genomic RNA, complete genome.	Norovirus Hu/GII-4/Niigata2/2008/JP	03-AUG-2010	302128658	7509
AB541313.1	Norovirus Hu/GII-4/Niigata3/2007/JP genomic RNA, complete genome.	Norovirus Hu/GII-4/Niigata3/2007/JP	03-AUG-2010	302128662	7509
AB541314.1	Norovirus Hu/GII-4/Niigata3/2008/JP genomic RNA, complete genome.	Norovirus Hu/GII-4/Niigata3/2008/JP	03-AUG-2010	302128666	7509
AB541315.1	Norovirus Hu/GII-4/Niigata4/2007/JP genomic RNA, complete genome.	Norovirus Hu/GII-4/Niigata4/2007/JP	03-AUG-2010	302128670	7509
AB541316.1	Norovirus Hu/GII-4/Niigata4/2008/JP genomic RNA, complete genome.	Norovirus Hu/GII-4/Niigata4/2008/JP	03-AUG-2010	302128674	7509
AB541317.1	Norovirus Hu/GII-4/Niigata5/2007/JP genomic RNA, complete genome.	Norovirus Hu/GII-4/Niigata5/2007/JP	03-AUG-2010	302128678	7509
AB541318.1	Norovirus Hu/GII-4/Niigata5/2008/JP genomic RNA, complete genome.	Norovirus Hu/GII-4/Niigata5/2008/JP	03-AUG-2010	302128682	7509
AB541319.1	Norovirus Hu/GII-4/Osaka1/2007/JP genomic RNA, complete genome.	Norovirus Hu/GII-4/Osaka1/2007/JP	03-AUG-2010	302128686	7509
AB541320.1	Norovirus Hu/GII-4/Osaka1/2008/JP genomic RNA, complete genome.	Norovirus Hu/GII-4/Osaka1/2008/JP	03-AUG-2010	302128690	7509

AB541321.1	Norovirus Hu/GII-4/Osaka2/2007/JP genomic RNA, complete genome.	Norovirus Hu/GII-4/Osaka2/2007/JP	03-AUG-2010	302128694	7509
AB541322.1	Norovirus Hu/GII-4/Osaka2/2008/JP genomic RNA, complete genome.	Norovirus Hu/GII-4/Osaka2/2008/JP	03-AUG-2010	302128698	7509
AB541323.1	Norovirus Hu/GII-4/Osaka3/2007/JP genomic RNA, complete genome.	Norovirus Hu/GII-4/Osaka3/2007/JP	03-AUG-2010	302128702	7509
AB541324.1	Norovirus Hu/GII-4/Osaka3/2008/JP genomic RNA, complete genome.	Norovirus Hu/GII-4/Osaka3/2008/JP	03-AUG-2010	302128706	7509
AB541325.1	Norovirus Hu/GII-4/Osaka4/2007/JP genomic RNA, complete genome.	Norovirus Hu/GII-4/Osaka4/2007/JP	03-AUG-2010	302128710	7509
AB541326.1	Norovirus Hu/GII-4/Osaka4/2008/JP genomic RNA, complete genome.	Norovirus Hu/GII-4/Osaka4/2008/JP	03-AUG-2010	302128714	7509
AB541327.1	Norovirus Hu/GII-4/Osaka5/2007/JP genomic RNA, complete genome.	Norovirus Hu/GII-4/Osaka5/2007/JP	03-AUG-2010	302128718	7509
AB541328.1	Norovirus Hu/GII-4/Osaka5/2008/JP genomic RNA, complete genome.	Norovirus Hu/GII-4/Osaka5/2008/JP	03-AUG-2010	302128722	7509
AB541329.1	Norovirus Hu/GII-4/Osaka6/2008/JP genomic RNA, complete genome.	Norovirus Hu/GII-4/Osaka6/2008/JP	03-AUG-2010	302128726	7509
AB541330.1	Norovirus Hu/GII-4/Saga1/2007/JP genomic RNA, complete genome.	Norovirus Hu/GII-4/Saga1/2007/JP	03-AUG-2010	302128730	7509
AB541331.1	Norovirus Hu/GII-4/Saga1/2008/JP genomic RNA, complete genome.	Norovirus Hu/GII-4/Saga1/2008/JP	03-AUG-2010	302128734	7509
AB541332.1	Norovirus Hu/GII-4/Saga2/2007/JP genomic RNA, complete genome.	Norovirus Hu/GII-4/Saga2/2007/JP	03-AUG-2010	302128738	7509
AB541333.1	Norovirus Hu/GII-4/Saga2/2008/JP genomic RNA, complete genome.	Norovirus Hu/GII-4/Saga2/2008/JP	03-AUG-2010	302128742	7509
AB541334.1	Norovirus Hu/GII-4/Saga3/2008/JP genomic RNA, complete genome.	Norovirus Hu/GII-4/Saga3/2008/JP	03-AUG-2010	302128746	7509
AB541335.1	Norovirus Hu/GII-4/Saga4/2007/JP genomic RNA, complete genome.	Norovirus Hu/GII-4/Saga4/2007/JP	03-AUG-2010	302128750	7509
AB541336.1	Norovirus Hu/GII-4/Saga4/2008/JP genomic RNA, complete genome.	Norovirus Hu/GII-4/Saga4/2008/JP	03-AUG-2010	302128754	7509
AB541337.1	Norovirus Hu/GII-4/Saga5/2007/JP genomic RNA, complete genome.	Norovirus Hu/GII-4/Saga5/2007/JP	03-AUG-2010	302128758	7509
AB541338.1	Norovirus Hu/GII-4/Saga5/2008/JP genomic RNA, complete genome.	Norovirus Hu/GII-4/Saga5/2008/JP	03-AUG-2010	302128762	7509
AB541339.1	Norovirus Hu/GII-4/Sakai1/2007/JP genomic RNA, complete genome.	Norovirus Hu/GII-4/Sakai1/2007/JP	03-AUG-2010	302128766	7509
AB541340.1	Norovirus Hu/GII-4/Sakai1/2008/JP genomic RNA, complete genome.	Norovirus Hu/GII-4/Sakai1/2008/JP	03-AUG-2010	302128770	7509
AB541341.1	Norovirus Hu/GII-4/Sakai2/2007/JP genomic RNA, complete genome.	Norovirus Hu/GII-4/Sakai2/2007/JP	03-AUG-2010	302128774	7509
AB541342.1	Norovirus Hu/GII-4/Sakai3/2007/JP genomic RNA, complete genome.	Norovirus Hu/GII-4/Sakai3/2007/JP	03-AUG-2010	302128778	7509
AB541343.1	Norovirus Hu/GII-4/Sakai3/2008/JP genomic RNA, complete genome.	Norovirus Hu/GII-4/Sakai3/2008/JP	03-AUG-2010	302128782	7509
AB541344.1	Norovirus Hu/GII-4/Sakai4/2007/JP genomic RNA, complete genome.	Norovirus Hu/GII-4/Sakai4/2007/JP	03-AUG-2010	302128786	7509
AB541345.1	Norovirus Hu/GII-4/Sakai4/2008/JP genomic RNA, complete genome.	Norovirus Hu/GII-4/Sakai4/2008/JP	03-AUG-2010	302128790	7509
AB541346.1	Norovirus Hu/GII-4/Shimane1/2007/JP genomic RNA, complete genome.	Norovirus Hu/GII-4/Shimane1/2007/JP	03-AUG-2010	302128794	7509
AB541347.1	Norovirus Hu/GII-4/Shimane2/2007/JP genomic RNA, complete genome.	Norovirus Hu/GII-4/Shimane2/2007/JP	03-AUG-2010	302128798	7509
AB541348.1	Norovirus Hu/GII-4/Shimane2/2008/JP genomic RNA, nearly complete genome.	Norovirus Hu/GII-4/Shimane2/2008/JP	03-AUG-2010	302128802	7401
AB541349.1	Norovirus Hu/GII-4/Shimane3/2007/JP genomic RNA, complete genome.	Norovirus Hu/GII-4/Shimane3/2007/JP	03-AUG-2010	302128806	7509
AB541350.1	Norovirus Hu/GII-4/Shimane3/2008/JP genomic RNA, complete genome.	Norovirus Hu/GII-4/Shimane3/2008/JP	03-AUG-2010	302128810	7509
AB541351.1	Norovirus Hu/GII-4/Shimane4/2007/JP genomic RNA, complete genome.	Norovirus Hu/GII-4/Shimane4/2007/JP	03-AUG-2010	302128814	7509
AB541352.1	Norovirus Hu/GII-4/Shimane5/2007/JP genomic RNA, complete genome.	Norovirus Hu/GII-4/Shimane5/2007/JP	03-AUG-2010	302128818	7509
AB541353.1	Norovirus Hu/GII-4/Shimane5/2008/JP genomic RNA, complete genome.	Norovirus Hu/GII-4/Shimane5/2008/JP	03-AUG-2010	302128822	7509
AB541354.1	Norovirus Hu/GII-4/Toyama1/2007/JP genomic RNA, complete genome.	Norovirus Hu/GII-4/Toyama1/2007/JP	03-AUG-2010	302128826	7509
AB541355.1	Norovirus Hu/GII-4/Toyama2/2007/JP genomic RNA, complete genome.	Norovirus Hu/GII-4/Toyama2/2007/JP	03-AUG-2010	302128830	7509

AB541356.1	Norovirus Hu/GII-4/Toyama2/2008/JP genomic RNA, complete genome.	Norovirus Hu/GII-4/Toyama2/2008/JP	03-AUG-2010	302128834	7509
AB541357.1	Norovirus Hu/GII-4/Toyama3/2007/JP genomic RNA, complete genome.	Norovirus Hu/GII-4/Toyama3/2007/JP	03-AUG-2010	302128838	7512
AB541358.1	Norovirus Hu/GII-4/Toyama3/2008/JP genomic RNA, complete genome.	Norovirus Hu/GII-4/Toyama3/2008/JP	03-AUG-2010	302128842	7509
AB541359.1	Norovirus Hu/GII-4/Toyama4/2007/JP genomic RNA, complete genome.	Norovirus Hu/GII-4/Toyama4/2007/JP	03-AUG-2010	302128846	7509
AB541360.1	Norovirus Hu/GII-4/Toyama4/2008/JP genomic RNA, complete genome.	Norovirus Hu/GII-4/Toyama4/2008/JP	03-AUG-2010	302128850	7509
AB541361.1	Norovirus Hu/GII-4/Toyama5/2007/JP genomic RNA, complete genome.	Norovirus Hu/GII-4/Toyama5/2007/JP	03-AUG-2010	302128854	7509
AB541362.1	Norovirus Hu/GII-4/Toyama5/2008/JP genomic RNA, complete genome.	Norovirus Hu/GII-4/Toyama5/2008/JP	03-AUG-2010	302128858	7509
AB543808.1	Norovirus Hu/GII-4/FUMI/2010/JP genomic RNA, complete genome.	Norovirus Hu/GII-4/FUMI/2010/JP	16-FEB-2010	288872177	7509
GU445325.2	Norovirus Hu/GII.4/New Orleans1805/2009/USA, complete genome.	Norovirus Hu/GII.4/New Orleans1805/2009	18-AUG-2011	343796574	7559
GU594162.1	Norovirus Hu/GII/8610/Saga/2008/JPN, complete genome.	Norovirus Hu/GII/8610/Saga/2008/JPN	03-APR-2010	292486043	7529
GU991353.1	Norovirus Hu/GII/Shanghai/SH2/2008/CHN, complete genome.	Norovirus Hu/GII/Shanghai/SH2/2008/CHN	27-FEB-2012	294869028	7555
GU991354.1	Norovirus Hu/Shanghai/SH5/2009/CHN, complete genome.	Norovirus Hu/Shanghai/SH5/2009/CHN	27-FEB-2012	294869033	7511
GU991355.1	Norovirus Hu/Shanghai/SH312/2009/CHN, complete genome.	Norovirus Hu/Shanghai/SH312/2009/CHN	27-FEB-2012	294869037	7544
GU980585.1	Norovirus Hu/GII.3/CBNU1/2006/KOR, complete genome.	Norovirus Hu/GII.3/CBNU1/2006/KOR	10-AUG-2010	300492589	7577
HQ009513.1	Norovirus Hu/GII.4/JB-15/KOR/2008, complete genome.	Norovirus Hu/GII.4/JB-15/KOR/2008	05-SEP-2010	305415031	7558
HM748971.2	Norovirus Hu/GII.4/Beecroft/NSW305P/2009/AUS, complete genome.	Norovirus Hu/GII.4/Beecroft/NSW305P/20	06-FEB-2012	374674596	7560
HM748972.2	Norovirus Hu/GII.4/Teralba/NSW881Z/2009/AUS, complete genome.	Norovirus Hu/GII.4/Teralba/NSW881Z/200	06-FEB-2012	374674599	7559
HM748973.2	Norovirus Hu/GII.4/Turramurra/NSW892U/2009/AUS, complete genome.	Norovirus Hu/GII.4/Turramurra/NSW892U	06-FEB-2012	374674602	7560
HQ449728.1	Norovirus Hu/GII.12/HS210/2010/USA, complete genome.	Norovirus Hu/GII.12/HS210/2010/USA	06-SEP-2011	315018888	7523
JF320644.1	Murine norovirus GV/NIH-2409/2005/USA, complete genome.	Murine norovirus GV/NIH-2409/2005/USA	09-MAY-2011	332143578	7383
JF320645.1	Murine norovirus GV/NIH-2410/2005/USA, complete genome.	Murine norovirus GV/NIH-2410/2005/USA	09-MAY-2011	332143582	7383
JF320646.1	Murine norovirus GV/NIH-2411/2005/USA, complete genome.	Murine norovirus GV/NIH-2411/2005/USA	09-MAY-2011	332143586	7383
JF320647.1	Murine norovirus GV/NIH-2747/2005/USA, complete genome.	Murine norovirus GV/NIH-2747/2005/USA	09-MAY-2011	332143590	7383
JF320648.1	Murine norovirus GV/NIH-2750/2005/USA, complete genome.	Murine norovirus GV/NIH-2750/2005/USA	09-MAY-2011	332143594	7383
JF320649.1	Murine norovirus GV/NIH-4421/2005/USA, complete genome.	Murine norovirus GV/NIH-4421/2005/USA	09-MAY-2011	332143598	7383
JF320650.1	Murine norovirus GV/NIH-4428/2005/USA, complete genome.	Murine norovirus GV/NIH-4428/2005/USA	09-MAY-2011	332143602	7383
JF320651.1	Murine norovirus GV/NIH-4431/2005/USA, complete genome.	Murine norovirus GV/NIH-4431/2005/USA	09-MAY-2011	332143606	7383
JF320652.1	Murine norovirus GV/NIH-A114/2006/USA, complete genome.	Murine norovirus GV/NIH-A114/2006/USA	09-MAY-2011	332143610	7382
JF320653.1	Murine norovirus GV/NIH-D220/2007/USA, complete genome.	Murine norovirus GV/NIH-D220/2007/USA	09-MAY-2011	332143614	7382
HQ664990.1	Norovirus Hu/GII.12/HS206/2010/USA, complete genome.	Norovirus Hu/GII.12/HS206/2010/USA	06-SEP-2011	336286250	7523
HQ392821.1	Norovirus pig/GII/Ch6/China/2009, complete genome.	Norovirus pig/GII/Ch6/China/2009	01-JUN-2012	347551769	7548
JN595867.1	Norovirus Hu/GII.4/New Orleans/2010/USA, complete genome.	Norovirus Hu/GII.4/New Orleans/2010/US/	22-JAN-2012	372292170	7559
JF781268.1	Norovirus cat/GIV.2/CU081210E/USA/2010, complete genome.	Norovirus cat/GIV.2/CU081210E/USA/201	19-MAR-2012	380036198	7839
JQ388274.1	Norovirus Hu/GI.6/Kingston/ACT160D/2010/AU, complete genome.	Norovirus Hu/GI.6/Kingston/ACT160D/201	22-APR-2012	384383709	7691
JQ911594.1	Norovirus Hu/GI/10360/2010/VNM, complete genome.	Norovirus Hu/GI/10360/2010/VNM	20-SEP-2012	384562888	7696

JQ911595.1	Norovirus Hu/GII/10002/2009/VNM, complete genome.	Norovirus Hu/GII/10002/2009/VNM	20-SEP-2012	384562892	7511
JQ911596.1	Norovirus Hu/GII/10003/2009/VNM, complete genome.	Norovirus Hu/GII/10003/2009/VNM	20-SEP-2012	384562896	7511
JQ911597.1	Norovirus Hu/GII/10012/2009/VNM, complete genome.	Norovirus Hu/GII/10012/2009/VNM	20-SEP-2012	384562900	7510
JQ911598.1	Norovirus Hu/GII/10037/2009/VNM, complete genome.	Norovirus Hu/GII/10037/2009/VNM	20-SEP-2012	384562904	7511
JQ613552.2	Norovirus Hu/GII.4/NSW123B/2010/AU, complete genome.	Norovirus Hu/GII.4/NSW123B/2010/AU	02-AUG-2012	399163231	7559
JQ613570.1	Norovirus Hu/GII.4/Rockdale/NSW006D/2009/AU, complete genome.	Norovirus Hu/GII.4/Rockdale/NSW006D/2	13-MAY-2012	386688515	7559
JQ613567.1	Norovirus Hu/GIV.1/LakeMacquarie/NSW268O/2010/AU, complete genome.	Norovirus Hu/GIV.1/LakeMacquarie/NSW2	24-AUG-2012	386688503	7527
JQ613569.1	Norovirus Hu/GII.g-GII.12/Gunnedah/NSW895P/2010/AU, complete genome.	Norovirus Hu/GII.g-GII.12/Gunnedah/NSW	13-MAY-2012	386688507	7523
JQ613568.1	Norovirus Hu/GII.g-GII.12/Wahroonga/NSW004P/2009/AU, complete genome.	Norovirus Hu/GII.g-GII.12/Wahroonga/NS\	13-MAY-2012	386688511	7523
JQ622197.1	Norovirus Hu/GII-4/CBNU2/2007/KR, complete genome.	Norovirus Hu/GII-4/CBNU2/2007/KR	07-NOV-2012	386783964	7583
JX023286.1	Norovirus Hu/GII.4/CHDC5191/1974/USA, complete genome.	Norovirus Hu/GII.4/CHDC5191/1974/USA	20-SEP-2012	386801118	7549
JX023285.1	Norovirus Hu/GI.1/8FIIa/1968/USA, complete genome.	Norovirus Hu/GI.1/8FIIa/1968/USA	20-SEP-2012	386801114	7633
JN400599.1	Norovirus Hu/GII-4/CGMH01/2006/TW, complete genome.	Norovirus Hu/GII-4/CGMH01/2006/TW	08-JUL-2012	393661138	7509
JN400600.1	Norovirus Hu/GII-4/CGMH02/2006/TW, complete genome.	Norovirus Hu/GII-4/CGMH02/2006/TW	08-JUL-2012	393661142	7509
JN400601.1	Norovirus Hu/GII-4/CGMH03/2006/TW, complete genome.	Norovirus Hu/GII-4/CGMH03/2006/TW	08-JUL-2012	393661146	7509
JN400602.1	Norovirus Hu/GII-4/CGMH04/2006/TW, complete genome.	Norovirus Hu/GII-4/CGMH04/2006/TW	08-JUL-2012	393661150	7509
JN400603.1	Norovirus Hu/GII-4/CGMH05/2006/TW, complete genome.	Norovirus Hu/GII-4/CGMH05/2006/TW	08-JUL-2012	393661154	7509
JN400604.1	Norovirus Hu/GII-4/CGMH06/2006/TW, complete genome.	Norovirus Hu/GII-4/CGMH06/2006/TW	08-JUL-2012	393661158	7509
JN400605.1	Norovirus Hu/GII-4/CGMH07/2006/TW, complete genome.	Norovirus Hu/GII-4/CGMH07/2006/TW	08-JUL-2012	393661162	7509
JN400606.1	Norovirus Hu/GII-4/CGMH08/2006/TW, complete genome.	Norovirus Hu/GII-4/CGMH08/2006/TW	08-JUL-2012	393661166	7509
JN400607.1	Norovirus Hu/GII-4/CGMH09/2006/TW, complete genome.	Norovirus Hu/GII-4/CGMH09/2006/TW	08-JUL-2012	393661170	7509
JN400608.1	Norovirus Hu/GII-4/CGMH10/2006/TW, complete genome.	Norovirus Hu/GII-4/CGMH10/2006/TW	08-JUL-2012	393661174	7509
JN400609.1	Norovirus Hu/GII-4/CGMH11/2006/TW, complete genome.	Norovirus Hu/GII-4/CGMH11/2006/TW	08-JUL-2012	393661178	7509
JN400610.1	Norovirus Hu/GII-4/CGMH12/2007/TW, complete genome.	Norovirus Hu/GII-4/CGMH12/2007/TW	08-JUL-2012	393661182	7509
JN400611.1	Norovirus Hu/GII-4/CGMH13/2007/TW, complete genome.	Norovirus Hu/GII-4/CGMH13/2007/TW	08-JUL-2012	393661186	7509
JN400612.1	Norovirus Hu/GII-4/CGMH14/2007/TW, complete genome.	Norovirus Hu/GII-4/CGMH14/2007/TW	08-JUL-2012	393661190	7509
JN400613.1	Norovirus Hu/GII-4/CGMH15/2007/TW, complete genome.	Norovirus Hu/GII-4/CGMH15/2007/TW	08-JUL-2012	393661194	7509
JN400614.1	Norovirus Hu/GII-4/CGMH16/2007/TW, complete genome.	Norovirus Hu/GII-4/CGMH16/2007/TW	08-JUL-2012	393661198	7509
JN400615.1	Norovirus Hu/GII-4/CGMH17/2007/TW, complete genome.	Norovirus Hu/GII-4/CGMH17/2007/TW	08-JUL-2012	393661202	7509
JN400616.1	Norovirus Hu/GII-4/CGMH18/2008/TW, complete genome.	Norovirus Hu/GII-4/CGMH18/2008/TW	08-JUL-2012	393661206	7509
JN400617.1	Norovirus Hu/GII-4/CGMH19/2009/TW, complete genome.	Norovirus Hu/GII-4/CGMH19/2009/TW	08-JUL-2012	393661210	7509
JN400618.1	Norovirus Hu/GII-4/CGMH20/2009/TW, complete genome.	Norovirus Hu/GII-4/CGMH20/2009/TW	08-JUL-2012	393661214	7476
JN400619.1	Norovirus Hu/GII-4/CGMH21/2010/TW, complete genome.	Norovirus Hu/GII-4/CGMH21/2010/TW	08-JUL-2012	393661218	7500
JN400620.1	Norovirus Hu/GII-4/CGMH22/2010/TW, complete genome.	Norovirus Hu/GII-4/CGMH22/2010/TW	08-JUL-2012	393661222	7509
JN400621.1	Norovirus Hu/GII-4/CGMH23/2010/TW, complete genome.	Norovirus Hu/GII-4/CGMH23/2010/TW	08-JUL-2012	393661226	7509

JN400622.1	Norovirus Hu/GII-4/CGMH24/2010/TW, complete genome.	Norovirus Hu/GII-4/CGMH24/2010/TW	08-JUL-2012	393661230	7509
JN400623.1	Norovirus Hu/GII-4/CGMH25/2010/TW, complete genome.	Norovirus Hu/GII-4/CGMH25/2010/TW	08-JUL-2012	393661234	7509
JN400624.1	Norovirus Hu/GII-4/CGMH26/2010/TW, complete genome.	Norovirus Hu/GII-4/CGMH26/2010/TW	08-JUL-2012	393661238	7509
JN400625.1	Norovirus Hu/GII-4/CGMH27/2010/TW, complete genome.	Norovirus Hu/GII-4/CGMH27/2010/TW	08-JUL-2012	393661242	7509
JN400626.1	Norovirus Hu/GII-4/CGMH28/2010/TW, complete genome.	Norovirus Hu/GII-4/CGMH28/2010/TW	08-JUL-2012	393661246	7509
JQ798158.1	Norovirus Hu/GII.4/5M/USA/2004, complete genome.	Norovirus Hu/GII.4/5M/USA/2004	23-AUG-2012	390195622	7558
JX126913.1	Norovirus Hu/GII.4/Ohio/7G/2012/USA, complete genome.	Norovirus Hu/GII.4/Ohio/7G/2012/USA	20-AUG-2012	401021919	7559
JX126912.1	Norovirus Hu/GII.4/Ohio/7I/2012/USA, complete genome.	Norovirus Hu/GII.4/Ohio/7I/2012/USA	20-AUG-2012	401021915	7558
JX459902.1	Norovirus Hu/GII.4/Berowra/NSW767L/2012/AU, complete genome.	Norovirus Hu/GII.4/Berowra/NSW767L/20	21-OCT-2012	409032907	7559
JX459901.1	Norovirus Hu/GII.4/Caringbah/NSW409G/2011/AU, complete genome.	Norovirus Hu/GII.4/Caringbah/NSW409G/	21-OCT-2012	409032903	7559
JX459904.1	Norovirus Hu/GII.4/Doonside/NSW536I/2011/AU, complete genome.	Norovirus Hu/GII.4/Doonside/NSW536I/20	21-OCT-2012	409032915	7559
JX459903.1	Norovirus Hu/GII.4/Jannali/NSW774M/2011/AU, complete genome.	Norovirus Hu/GII.4/Jannali/NSW774M/201	21-OCT-2012	409032911	7559
JX459906.1	Norovirus Hu/GII.4/Miranda/NSW850K/2011/AU, complete genome.	Norovirus Hu/GII.4/Miranda/NSW850K/20	21-OCT-2012	409032923	7560
JX459900.1	Norovirus Hu/GII.4/Randwick/NSW882J/2011/AU, complete genome.	Norovirus Hu/GII.4/Randwick/NSW882J/2	21-OCT-2012	409032899	7558
JX459905.1	Norovirus Hu/GII.4/Randwick/NSW938K/2011/AU, complete genome.	Norovirus Hu/GII.4/Randwick/NSW938K/2	21-OCT-2012	409032919	7560
JX459908.1	Norovirus Hu/GII.4/Sydney/NSW0514/2012/AU, complete genome.	Norovirus Hu/GII.4/Sydney/NSW0514/201	21-OCT-2012	409032931	7564
JX459907.1	Norovirus Hu/GII.4/Woonona/NSW3309/2012/AU, complete genome.	Norovirus Hu/GII.4/Woonona/NSW3309/2	21-OCT-2012	409032927	7560
JX486101.1	Norovirus Rn/GV/HKU_CT2/HKG/2011 polyprotein gene, partial cds; and VP1 capsid protein and VP2 mi	Norovirus Rn/GV/HKU_CT2/HKG/2011	30-OCT-2012	409974167	7528
JX486102.1	Norovirus Rn/GV/HKU_KT/HKG/2012 polyprotein, VP1 capsid protein, and VP2 minor structural protein g	Norovirus Rn/GV/HKU_KT/HKG/2012	30-OCT-2012	409974171	7542
JX846924.1	Norovirus Hu/NLV/GII.3/Norwalk/HK71/1978/CHN, complete genome.	Norovirus Hu/NLV/GII.3/Norwalk/HK71/19	24-NOV-2012	421952699	7527
JX846927.1	Norovirus Hu/NLV/GII.9/Norwalk/CHDC4073/1984/USA, complete genome.	Norovirus Hu/NLV/GII.9/Norwalk/CHDC40	24-NOV-2012	421952711	7534
KC175342.1	Norovirus Hu/Norwalk/10034/2009/VNM, complete genome.	Norovirus Hu/Norwalk/10034/2009/VNM	25-NOV-2012	422313473	7511
KC175343.1	Norovirus Hu/Norwalk/10051/2009/VNM, complete genome.	Norovirus Hu/Norwalk/10051/2009/VNM	25-NOV-2012	422313477	7511
KC175344.1	Norovirus Hu/Norwalk/10054/2009/VNM, complete genome.	Norovirus Hu/Norwalk/10054/2009/VNM	25-NOV-2012	422313481	7511
KC175345.1	Norovirus Hu/Norwalk/10062/2009/VNM, complete genome.	Norovirus Hu/Norwalk/10062/2009/VNM	25-NOV-2012	422313485	7511
KC175346.1	Norovirus Hu/Norwalk/10074/2009/VNM, complete genome.	Norovirus Hu/Norwalk/10074/2009/VNM	25-NOV-2012	422313489	7511
KC175347.1	Norovirus Hu/Norwalk/10075/2009/VNM, complete genome.	Norovirus Hu/Norwalk/10075/2009/VNM	25-NOV-2012	422313493	7511
KC175348.1	Norovirus Hu/Norwalk/10078/2009/VNM, complete genome.	Norovirus Hu/Norwalk/10078/2009/VNM	25-NOV-2012	422313497	7511
KC175349.1	Norovirus Hu/Norwalk/10079/2009/VNM, complete genome.	Norovirus Hu/Norwalk/10079/2009/VNM	25-NOV-2012	422313501	7511
KC175350.1	Norovirus Hu/Norwalk/10110/2009/VNM, complete genome.	Norovirus Hu/Norwalk/10110/2009/VNM	25-NOV-2012	422313505	7511
KC175351.1	Norovirus Hu/Norwalk/10114/2009/VNM, complete genome.	Norovirus Hu/Norwalk/10114/2009/VNM	25-NOV-2012	422313509	7511
KC175352.1	Norovirus Hu/Norwalk/10116/2009/VNM, complete genome.	Norovirus Hu/Norwalk/10116/2009/VNM	25-NOV-2012	422313513	7511
KC175353.1	Norovirus Hu/Norwalk/10129/2009/VNM, complete genome.	Norovirus Hu/Norwalk/10129/2009/VNM	25-NOV-2012	422313517	7511
KC175354.1	Norovirus Hu/Norwalk/10136/2009/VNM, complete genome.	Norovirus Hu/Norwalk/10136/2009/VNM	25-NOV-2012	422313521	7511
KC175355.1	Norovirus Hu/Norwalk/10137/2009/VNM, complete genome.	Norovirus Hu/Norwalk/10137/2009/VNM	25-NOV-2012	422313525	7511

KC175356.1	Norovirus Hu/Norwalk/10145/2009/VNM, complete genome.	Norovirus Hu/Norwalk/10145/2009/VNM	25-NOV-2012	422313529	7511
KC175357.1	Norovirus Hu/Norwalk/10148/2009/VNM, complete genome.	Norovirus Hu/Norwalk/10148/2009/VNM	25-NOV-2012	422313533	7511
KC175358.1	Norovirus Hu/Norwalk/10158/2009/VNM, complete genome.	Norovirus Hu/Norwalk/10158/2009/VNM	25-NOV-2012	422313537	7511
KC175359.1	Norovirus Hu/Norwalk/10160/2009/VNM, complete genome.	Norovirus Hu/Norwalk/10160/2009/VNM	25-NOV-2012	422313541	7511
KC175360.1	Norovirus Hu/Norwalk/10162/2009/VNM, complete genome.	Norovirus Hu/Norwalk/10162/2009/VNM	25-NOV-2012	422313545	7511
KC175361.1	Norovirus Hu/Norwalk/10163/2009/VNM, complete genome.	Norovirus Hu/Norwalk/10163/2009/VNM	25-NOV-2012	422313549	7511
KC175362.1	Norovirus Hu/Norwalk/10169/2009/VNM, complete genome.	Norovirus Hu/Norwalk/10169/2009/VNM	25-NOV-2012	422313553	7511
KC175363.1	Norovirus Hu/Norwalk/10173/2009/VNM, complete genome.	Norovirus Hu/Norwalk/10173/2009/VNM	25-NOV-2012	422313557	7511
KC175364.1	Norovirus Hu/Norwalk/10176/2009/VNM, complete genome.	Norovirus Hu/Norwalk/10176/2009/VNM	25-NOV-2012	422313561	7511
KC175365.1	Norovirus Hu/Norwalk/10177/2009/VNM, complete genome.	Norovirus Hu/Norwalk/10177/2009/VNM	25-NOV-2012	422313565	7511
KC175366.1	Norovirus Hu/Norwalk/10179/2009/VNM, complete genome.	Norovirus Hu/Norwalk/10179/2009/VNM	25-NOV-2012	422313569	7511
KC175367.1	Norovirus Hu/Norwalk/10182/2009/VNM, complete genome.	Norovirus Hu/Norwalk/10182/2009/VNM	25-NOV-2012	422313573	7511
KC175368.1	Norovirus Hu/Norwalk/10183/2009/VNM, complete genome.	Norovirus Hu/Norwalk/10183/2009/VNM	25-NOV-2012	422313577	7511
KC175369.1	Norovirus Hu/Norwalk/10194/2009/VNM, complete genome.	Norovirus Hu/Norwalk/10194/2009/VNM	25-NOV-2012	422313581	7511
KC175370.1	Norovirus Hu/Norwalk/10199/2009/VNM, complete genome.	Norovirus Hu/Norwalk/10199/2009/VNM	25-NOV-2012	422313585	7511
KC175371.1	Norovirus Hu/Norwalk/10203/2009/VNM, complete genome.	Norovirus Hu/Norwalk/10203/2009/VNM	25-NOV-2012	422313589	7511
KC175372.1	Norovirus Hu/Norwalk/10204/2009/VNM, complete genome.	Norovirus Hu/Norwalk/10204/2009/VNM	25-NOV-2012	422313593	7511
KC175373.1	Norovirus Hu/Norwalk/10222/2009/VNM, complete genome.	Norovirus Hu/Norwalk/10222/2009/VNM	25-NOV-2012	422313597	7511
KC175374.1	Norovirus Hu/Norwalk/10223/2009/VNM, complete genome.	Norovirus Hu/Norwalk/10223/2009/VNM	25-NOV-2012	422313601	7511
KC175375.1	Norovirus Hu/Norwalk/10235/2009/VNM, complete genome.	Norovirus Hu/Norwalk/10235/2009/VNM	25-NOV-2012	422313605	7511
KC175376.1	Norovirus Hu/Norwalk/10236/2009/VNM, complete genome.	Norovirus Hu/Norwalk/10236/2009/VNM	25-NOV-2012	422313609	7511
KC175377.1	Norovirus Hu/Norwalk/10238/2009/VNM, complete genome.	Norovirus Hu/Norwalk/10238/2009/VNM	25-NOV-2012	422313613	7511
KC175378.1	Norovirus Hu/Norwalk/10247/2009/VNM, complete genome.	Norovirus Hu/Norwalk/10247/2009/VNM	25-NOV-2012	422313617	7511
KC175379.1	Norovirus Hu/Norwalk/10255/2009/VNM, complete genome.	Norovirus Hu/Norwalk/10255/2009/VNM	25-NOV-2012	422313621	7511
KC175380.1	Norovirus Hu/Norwalk/10285/2010/VNM, complete genome.	Norovirus Hu/Norwalk/10285/2010/VNM	25-NOV-2012	422313625	7511
KC175381.1	Norovirus Hu/Norwalk/10296/2010/VNM, complete genome.	Norovirus Hu/Norwalk/10296/2010/VNM	25-NOV-2012	422313629	7511
KC175382.1	Norovirus Hu/Norwalk/10313/2010/VNM, complete genome.	Norovirus Hu/Norwalk/10313/2010/VNM	25-NOV-2012	422313633	7511
KC175383.1	Norovirus Hu/Norwalk/10325/2010/VNM, complete genome.	Norovirus Hu/Norwalk/10325/2010/VNM	25-NOV-2012	422313637	7511
KC175384.1	Norovirus Hu/Norwalk/10328/2010/VNM, complete genome.	Norovirus Hu/Norwalk/10328/2010/VNM	25-NOV-2012	422313641	7511
KC175385.1	Norovirus Hu/Norwalk/10368/2010/VNM, complete genome.	Norovirus Hu/Norwalk/10368/2010/VNM	25-NOV-2012	422313645	7511
KC175386.1	Norovirus Hu/Norwalk/10378/2010/VNM, complete genome.	Norovirus Hu/Norwalk/10378/2010/VNM	25-NOV-2012	422313649	7511
KC175387.1	Norovirus Hu/Norwalk/10386/2010/VNM, complete genome.	Norovirus Hu/Norwalk/10386/2010/VNM	25-NOV-2012	422313653	7511
KC175388.1	Norovirus Hu/Norwalk/20008/2009/VNM, complete genome.	Norovirus Hu/Norwalk/20008/2009/VNM	25-NOV-2012	422313657	7511
KC175389.1	Norovirus Hu/Norwalk/20010/2009/VNM, complete genome.	Norovirus Hu/Norwalk/20010/2009/VNM	25-NOV-2012	422313661	7511
KC175390.1	Norovirus Hu/Norwalk/20014/2009/VNM, complete genome.	Norovirus Hu/Norwalk/20014/2009/VNM	25-NOV-2012	422313665	7511

KC175391.1	Norovirus Hu/Norwalk/20016/2009/VNM, complete genome.	Norovirus Hu/Norwalk/20016/2009/VNM	25-NOV-2012	422313669	7511
KC175392.1	Norovirus Hu/Norwalk/20019/2009/VNM, complete genome.	Norovirus Hu/Norwalk/20019/2009/VNM	25-NOV-2012	422313673	7511
KC175393.1	Norovirus Hu/Norwalk/20033/2009/VNM, complete genome.	Norovirus Hu/Norwalk/20033/2009/VNM	25-NOV-2012	422313677	7511
KC175394.1	Norovirus Hu/Norwalk/20035/2009/VNM, complete genome.	Norovirus Hu/Norwalk/20035/2009/VNM	25-NOV-2012	422313681	7511
KC175395.1	Norovirus Hu/Norwalk/20044/2009/VNM, complete genome.	Norovirus Hu/Norwalk/20044/2009/VNM	25-NOV-2012	422313685	7511
KC175396.1	Norovirus Hu/Norwalk/20047/2009/VNM, complete genome.	Norovirus Hu/Norwalk/20047/2009/VNM	25-NOV-2012	422313689	7511
KC175397.1	Norovirus Hu/Norwalk/20066/2009/VNM, complete genome.	Norovirus Hu/Norwalk/20066/2009/VNM	25-NOV-2012	422313693	7511
KC175398.1	Norovirus Hu/Norwalk/20067/2009/VNM, complete genome.	Norovirus Hu/Norwalk/20067/2009/VNM	25-NOV-2012	422313697	7511
KC175399.1	Norovirus Hu/Norwalk/20069/2009/VNM, complete genome.	Norovirus Hu/Norwalk/20069/2009/VNM	25-NOV-2012	422313701	7511
KC175400.1	Norovirus Hu/Norwalk/20092/2009/VNM, complete genome.	Norovirus Hu/Norwalk/20092/2009/VNM	25-NOV-2012	422313705	7511
KC175401.1	Norovirus Hu/Norwalk/20093/2009/VNM, complete genome.	Norovirus Hu/Norwalk/20093/2009/VNM	25-NOV-2012	422313709	7511
KC175402.1	Norovirus Hu/Norwalk/20094/2009/VNM, complete genome.	Norovirus Hu/Norwalk/20094/2009/VNM	25-NOV-2012	422313713	7511
KC175403.1	Norovirus Hu/Norwalk/20118/2009/VNM, complete genome.	Norovirus Hu/Norwalk/20118/2009/VNM	25-NOV-2012	422313717	7511
KC175404.1	Norovirus Hu/Norwalk/20122/2009/VNM, complete genome.	Norovirus Hu/Norwalk/20122/2009/VNM	25-NOV-2012	422313721	7511
KC175405.1	Norovirus Hu/Norwalk/20123/2009/VNM, complete genome.	Norovirus Hu/Norwalk/20123/2009/VNM	25-NOV-2012	422313725	7511
KC175406.1	Norovirus Hu/Norwalk/20128/2009/VNM, complete genome.	Norovirus Hu/Norwalk/20128/2009/VNM	25-NOV-2012	422313729	7511
KC175407.1	Norovirus Hu/Norwalk/20135/2009/VNM, complete genome.	Norovirus Hu/Norwalk/20135/2009/VNM	25-NOV-2012	422313733	7511
KC175408.1	Norovirus Hu/Norwalk/20139/2009/VNM, complete genome.	Norovirus Hu/Norwalk/20139/2009/VNM	25-NOV-2012	422313737	7511
KC175409.1	Norovirus Hu/Norwalk/20140/2009/VNM, complete genome.	Norovirus Hu/Norwalk/20140/2009/VNM	25-NOV-2012	422313741	7511
KC175410.1	Norovirus Hu/Norwalk/20142/2009/VNM, complete genome.	Norovirus Hu/Norwalk/20142/2009/VNM	25-NOV-2012	422313745	7511
KC175323.1	Norovirus Hu/GII.4/Hong Kong/CUHK3630/2012/CHN, complete genome.	Norovirus Hu/GII.4/Hong Kong/CUHK363	02-DEC-2012	425707214	7559
KC013592.1	Norovirus Hu/GII.4/HS191/2004/USA, complete genome.	Norovirus Hu/GII.4/HS191/2004/USA	16-DEC-2012	429346005	7556
